# Supplementary material for: New Piperazine Derivatives of 6-Acetyl-7-hydroxy-4-methylcoumarin as 5-HT1A Receptor Agents
Source: Int J Mol Sci. 2023 Feb 1;24(3):2779. doi: 10.3390/ijms24032779 (PMC9917830; doi:10.3390/ijms24032779)
Supplement: Supplementary file 1 [file ijms-24-02779-s001.zip › ijms-2187388-supplementary.pdf]

New Piperazine Derivatives of 6-Acetyl-7-hydroxy-4-methylcoumarin as 5-HT1A Receptor Agents

Kinga Ostrowska [1,*], Anna Leśniak [2], Weronika Gryczka [1], Łukasz Dobrzycki [3], Magdalena Bujalska-Zadrożny [2] and Bartosz Trzaskowski [4]

[1] Department of Organic and Physical Chemistry, Faculty of Pharmacy, Medical University of Warsaw, 1 Banacha Str., 02-097 Warsaw, Poland
[2] Centre for Preclincal Research and Technology, Department of Pharmacodynamics, Faculty of Pharmacy, Medical University of Warsaw, 1 Banacha Str., 02-97 Warsaw, Poland
[3] Crystallochemistry Laboratory, Chemistry Department, Warsaw University, 1 Pasteura Str., 02-093 Warsaw, Poland
[4] Centre of New Technologies, University of Warsaw, 2C Banacha Str., 02-097 Warsaw, Poland
* Correspondence: kostrowska@wum.edu.pl; Tel.: +48-22-572-0669

^{1}H NMR and ^{13}C NMR Spectra

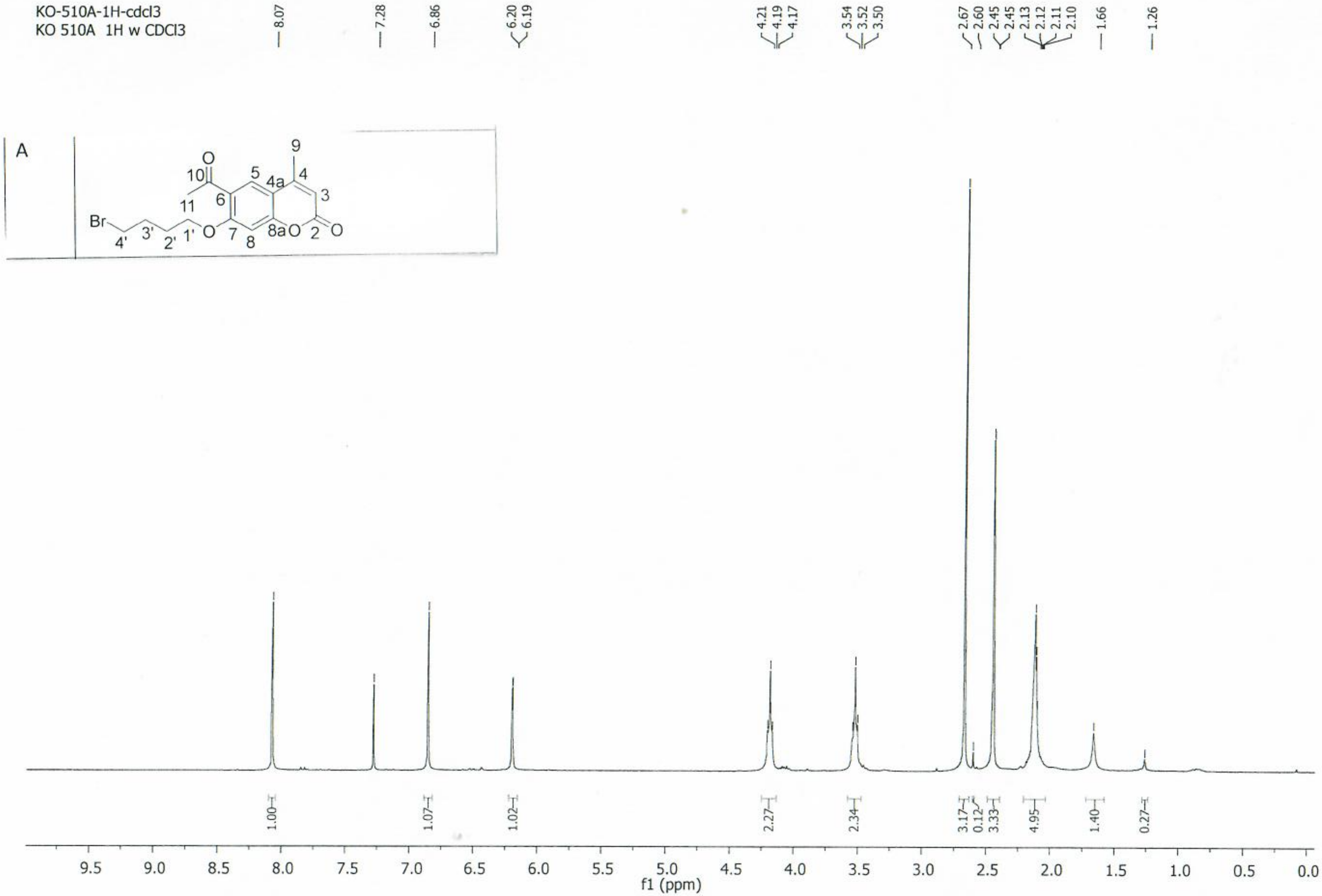
KO-510A-1H-cdcl3
KO 510A 1H w CDCl3
8.07
7.28
6.86
6.20
6.19
4.21
4.19
4.17
3.54
3.52
3.50
2.67
2.60
2.45
2.45
2.13
2.12
2.11
2.10
1.66
1.26
A
Br
O
1'
2'
3'
4'
4a
8a
10
11
9
1.00
1.07
1.02
2.27
2.34
3.17
0.12
3.33
4.95
1.40
0.27
9.5
9.0
8.5
8.0
7.5
7.0
6.5
6.0
5.5
5.0
4.5
4.0
3.5
3.0
2.5
2.0
1.5
1.0
0.5
0.0
f1 (ppm)

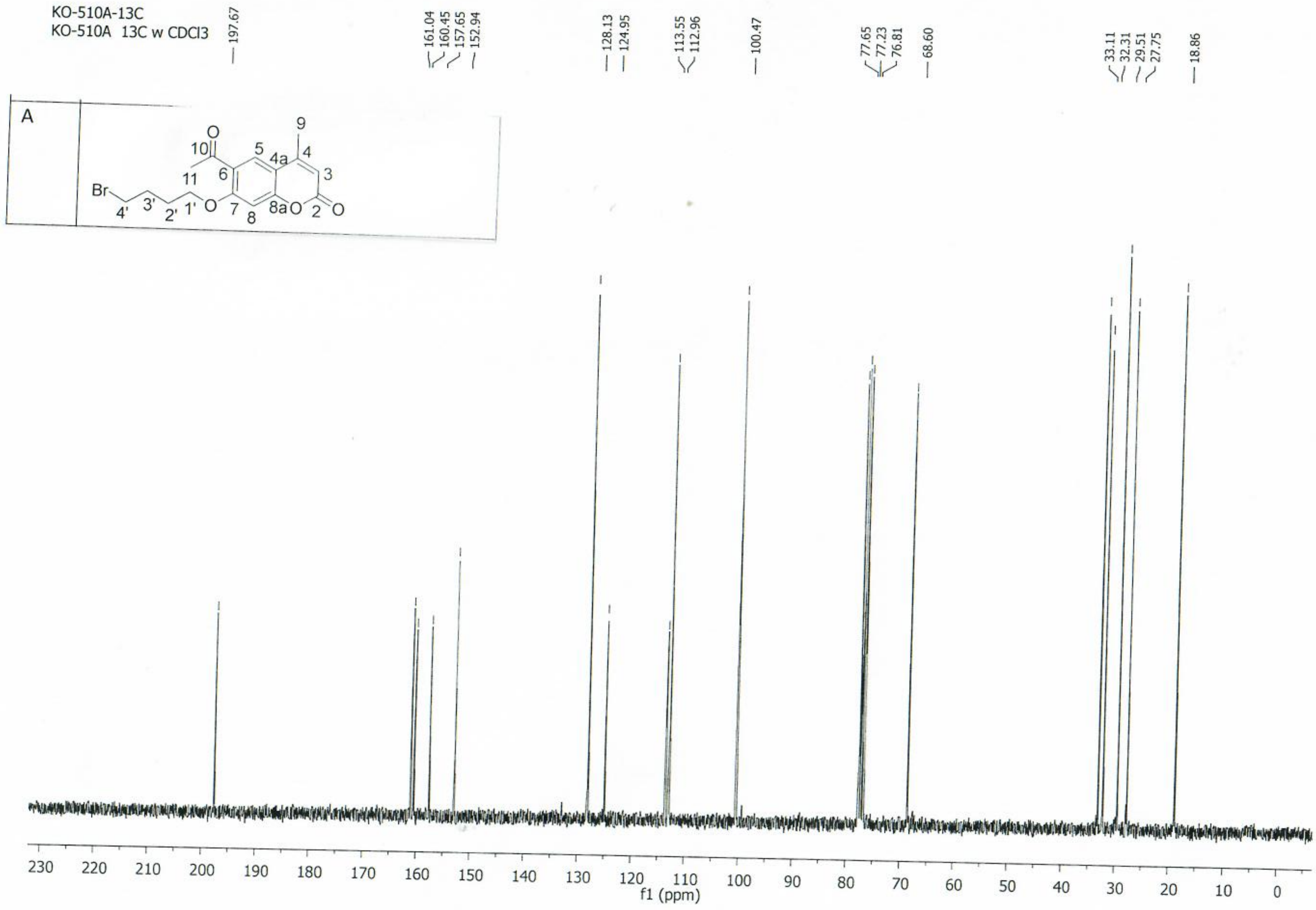

KO-510A-13C
KO-510A 13C w CDCl3
197.67
161.04
160.45
157.65
152.94
128.13
124.95
113.55
112.96
100.47
77.65
77.23
76.81
68.60
33.11
32.31
29.51
27.75
18.86
A
9
O
10
5
4a
4
3
11
6
Br
4'
3'
2'
1'
O
7
8
8a
O
2
O
230
220
210
200
190
180
170
160
150
140
130
120
110
100
90
80
70
60
50
40
30
20
10
0
f1 (ppm)

KO-511-I-1H-cdcl3
KO 511 I 1H w CDCl3

1

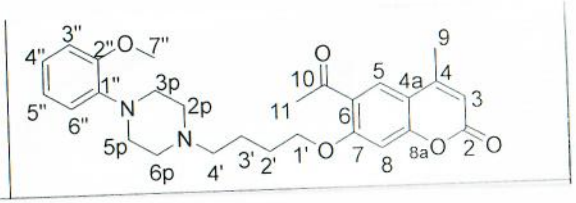

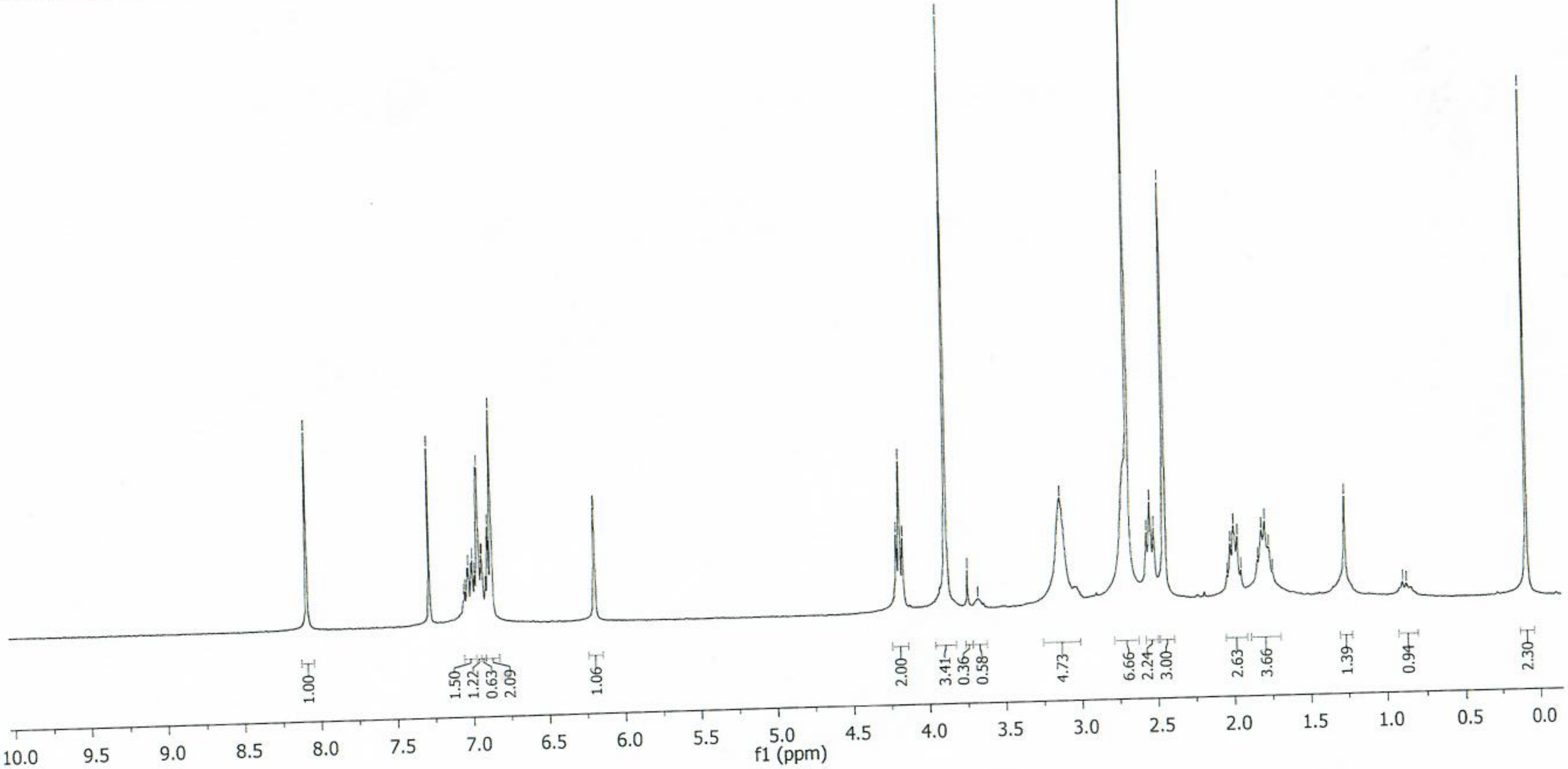

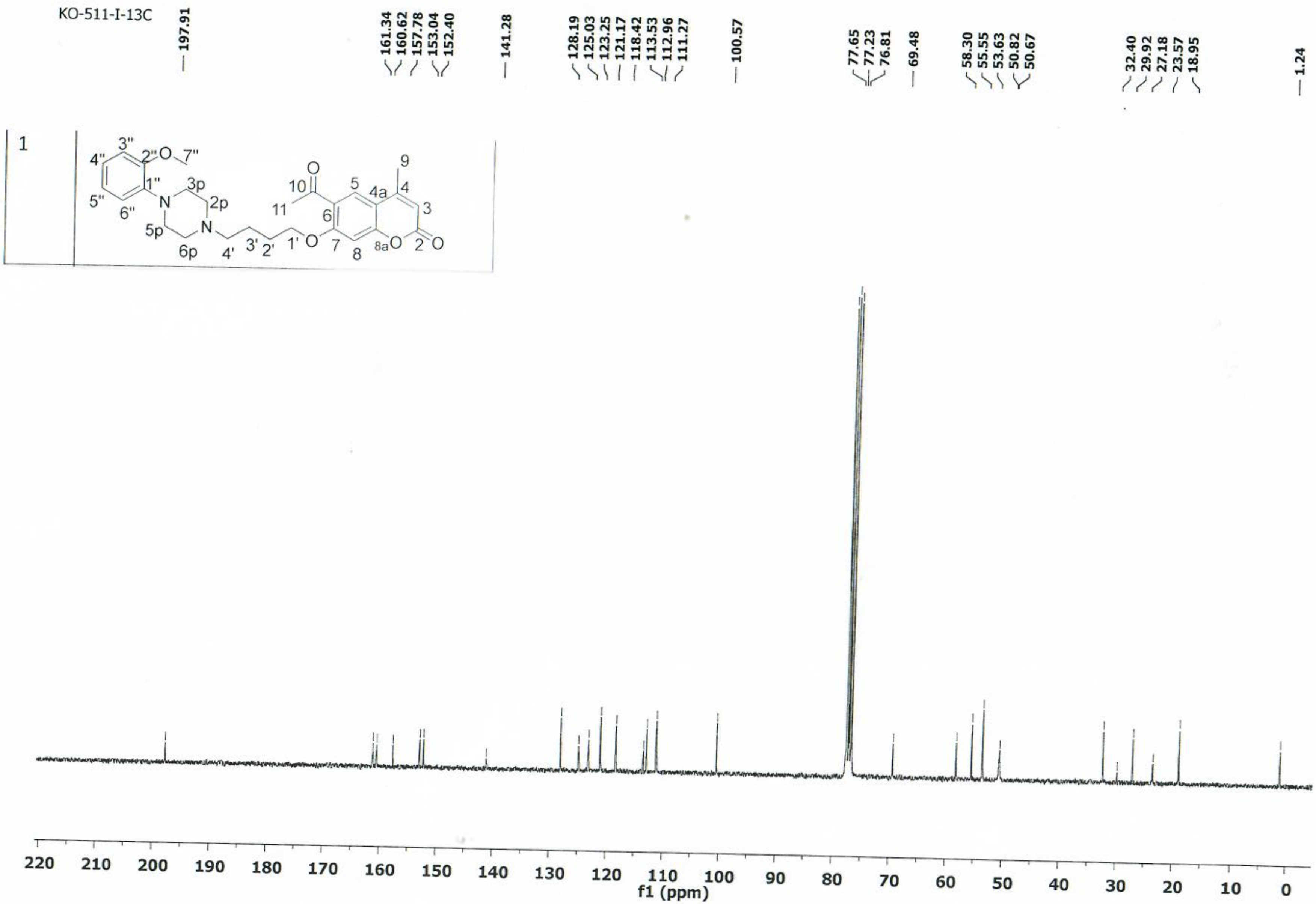
KO-511-I-13C
197.91
161.34
160.62
157.78
153.04
152.40
141.28
128.19
125.03
123.25
121.17
118.42
113.53
112.96
111.27
100.57
77.65
77.23
76.81
69.48
58.30
55.55
53.63
50.82
50.67
32.40
29.92
27.18
23.57
18.95
1.24
1
220 210 200 190 180 170 160 150 140 130 120 110 100 90 80 70 60 50 40 30 20 10 0
f1 (ppm)

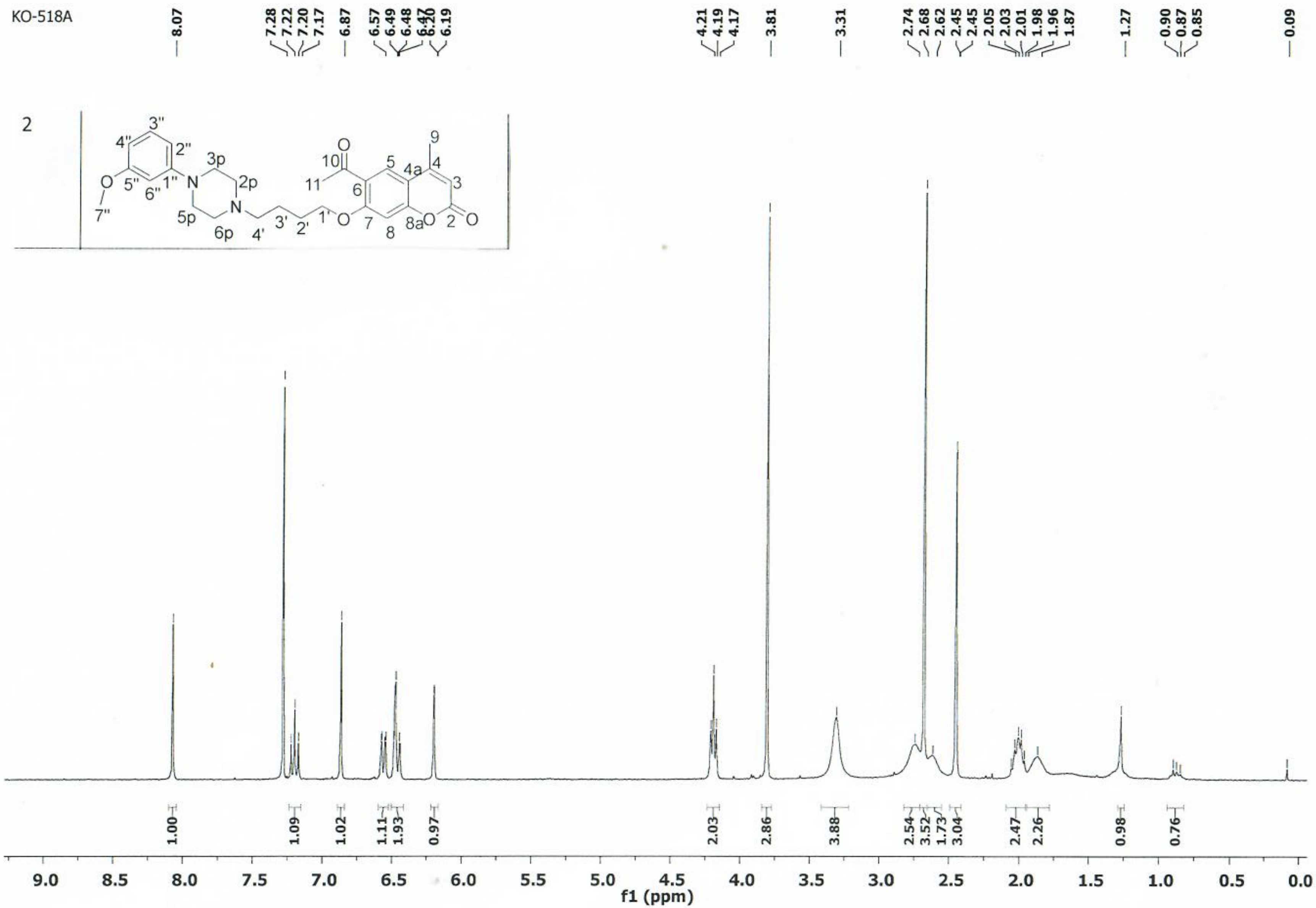
KO-518A
8.07
7.28
7.22
7.20
7.17
6.87
6.57
6.49
6.48
6.47
6.20
6.19
4.21
4.19
4.17
3.81
3.31
2.74
2.68
2.62
2.45
2.45
2.05
2.03
2.01
1.98
1.96
1.87
1.27
0.90
0.87
0.85
0.09
2
1.00
1.09
1.02
1.11
1.93
0.97
2.03
2.86
3.88
2.54
3.52
1.73
3.04
2.47
2.26
0.98
0.76
9.0 8.5 8.0 7.5 7.0 6.5 6.0 5.5 5.0 4.5 4.0 3.5 3.0 2.5 2.0 1.5 1.0 0.5 0.0
f1 (ppm)

KO-518A-13C

2

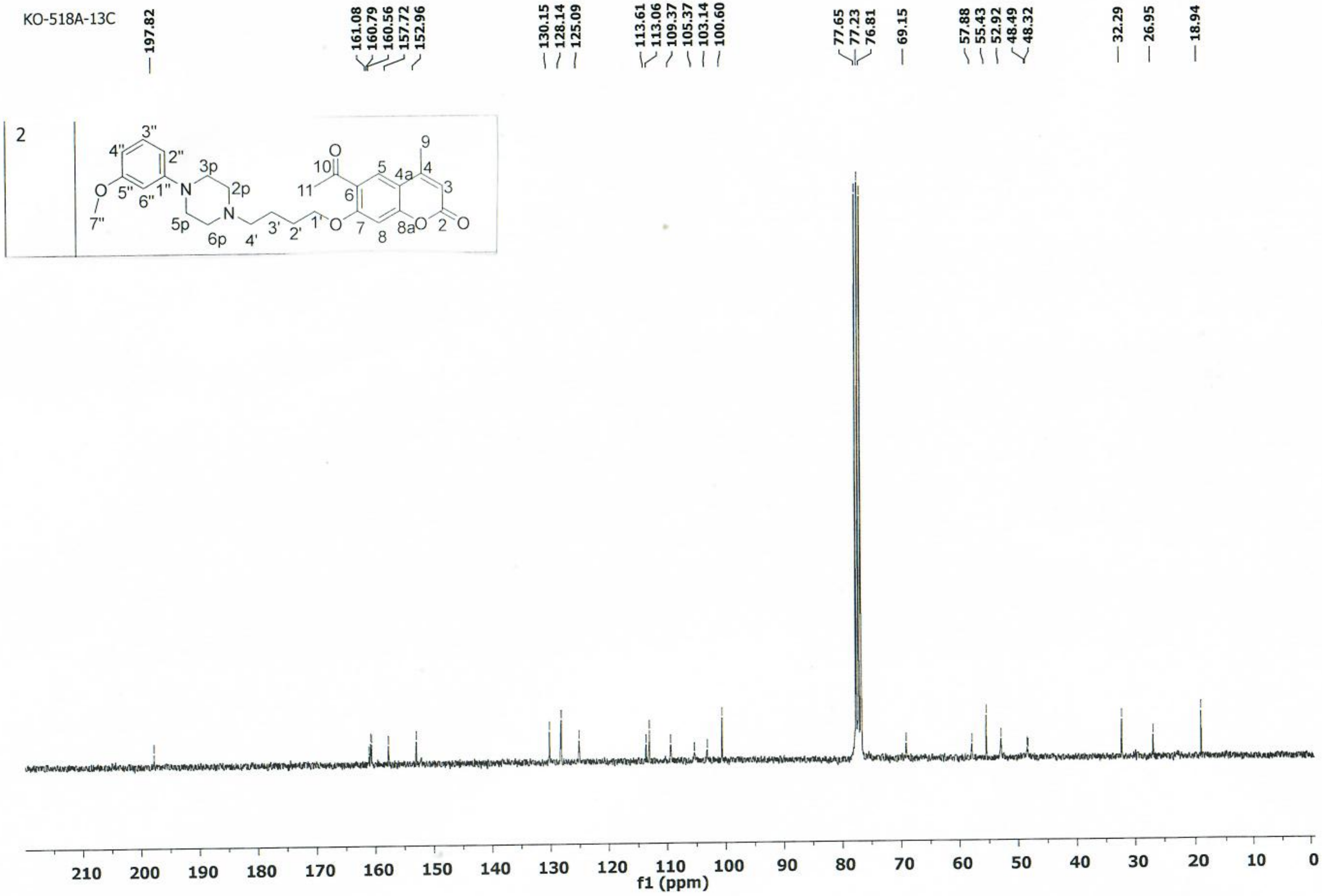
197.82
161.08
160.79
160.56
157.72
152.96
130.15
128.14
125.09
113.61
113.06
109.37
105.37
103.14
100.60
77.65
77.23
76.81
69.15
57.88
55.43
52.92
48.49
48.32
32.29
26.95
18.94
3''
4''
2''
3p
5''
6''
1''
2p
N
O
7''
5p
6p
4'
3'
2'
1'
O
10
11
6
5
4a
4
9
3
7
8
8a
2
210
200
190
180
170
160
150
140
130
120
110
100
90
80
70
60
50
40
30
20
10
0
f1 (ppm)

3

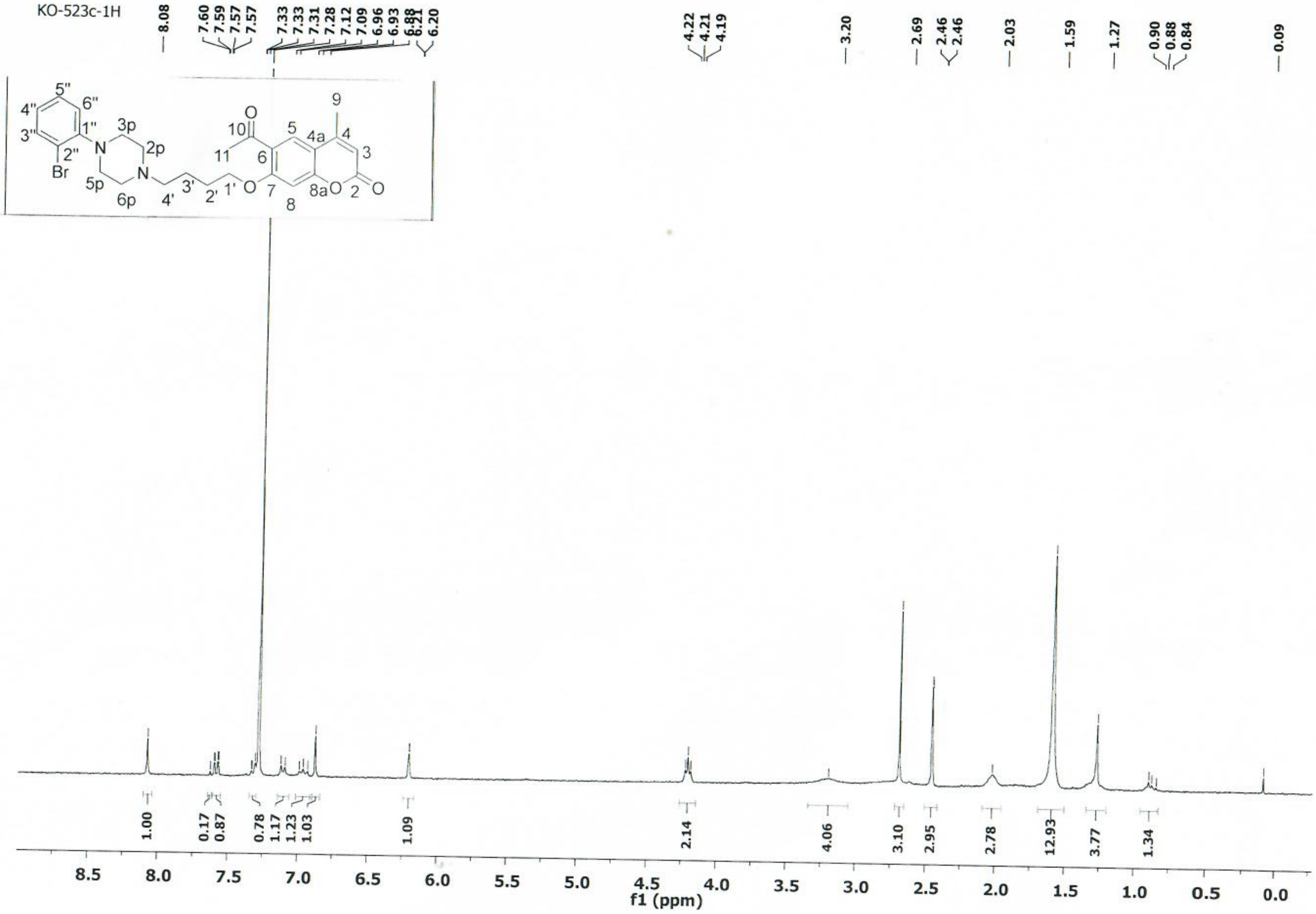
KO-523c-1H
8.08
7.60 7.59 7.57 7.57
7.33 7.33 7.31 7.28 7.12 7.09 6.96 6.93 6.88 6.21 6.20
4.22 4.21 4.19
3.20
2.69
2.46 2.46
2.03
1.59
1.27
0.90 0.88 0.84
0.09
1.00 0.17 0.87 0.78 1.17 1.23 1.03 1.09 2.14 4.06 3.10 2.95 2.78 12.93 3.77 1.34
8.5 8.0 7.5 7.0 6.5 6.0 5.5 5.0 4.5 4.0 3.5 3.0 2.5 2.0 1.5 1.0 0.5 0.0
f1 (ppm)
5'' 6'' 4'' 1'' 3p 3'' 2p 2'' Br N N 5p 6p 4' 3' 2' 1' O 10 O 11 6 5 4a 4 9 3 7 8 8a O 2 O

KO-523c-13C

3

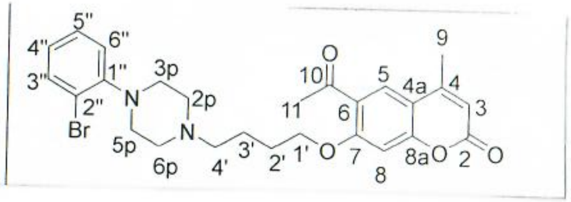
5''
6''
4''
3''
1''
2''
Br
3p
2p
N
N
5p
6p
4'
3'
2'
1'
O
7
8
8a
O
2
O
10
11
O
6
5
4a
4
9
3

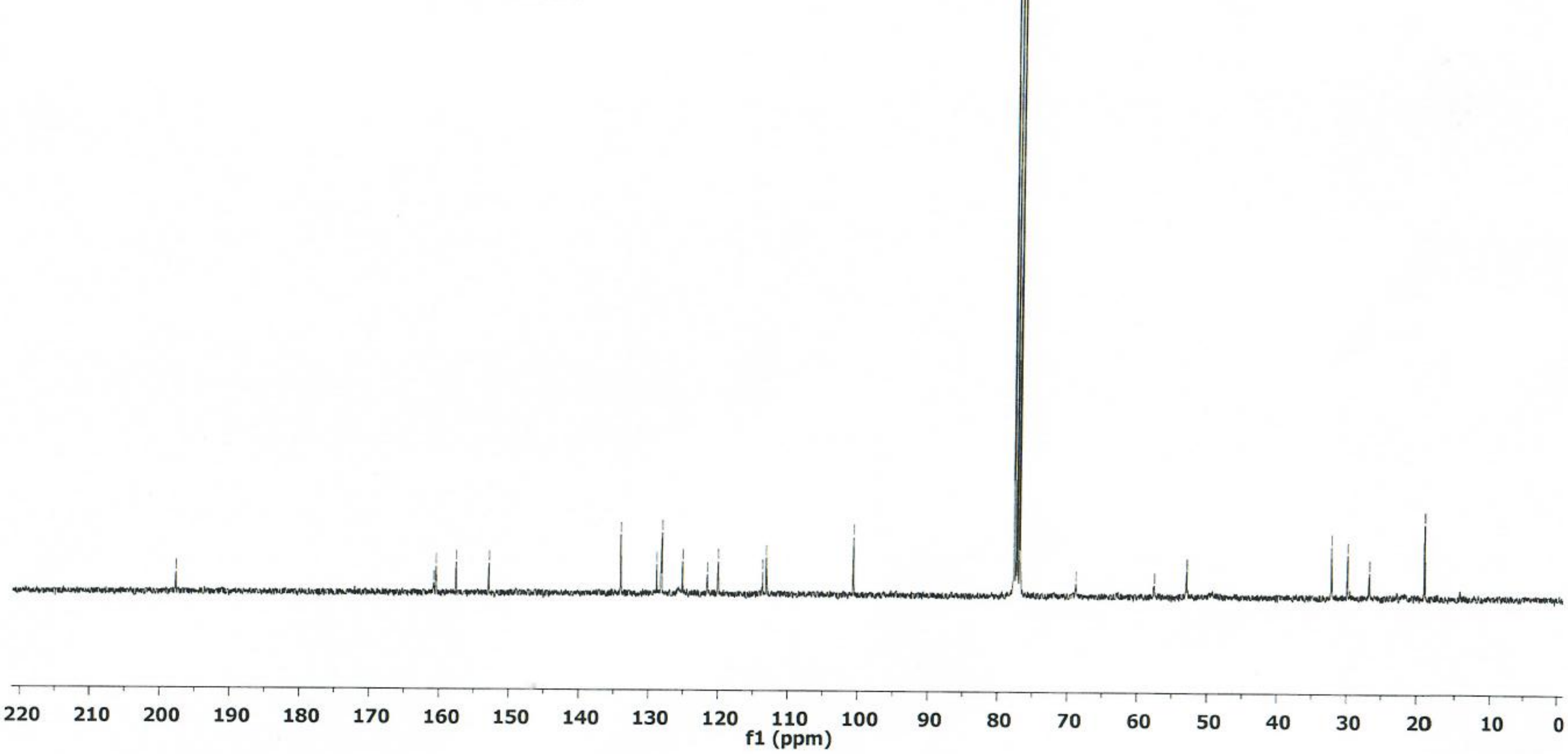
197.72
160.83
160.50
157.68
152.89
134.01
128.81
128.11
125.13
121.60
120.03
113.68
113.15
100.64
77.65
77.23
76.81
68.81
57.59
52.97
52.93
32.23
29.91
26.78
18.93
220 210 200 190 180 170 160 150 140 130 120 110 100 90 80 70 60 50 40 30 20 10 0
f1 (ppm)

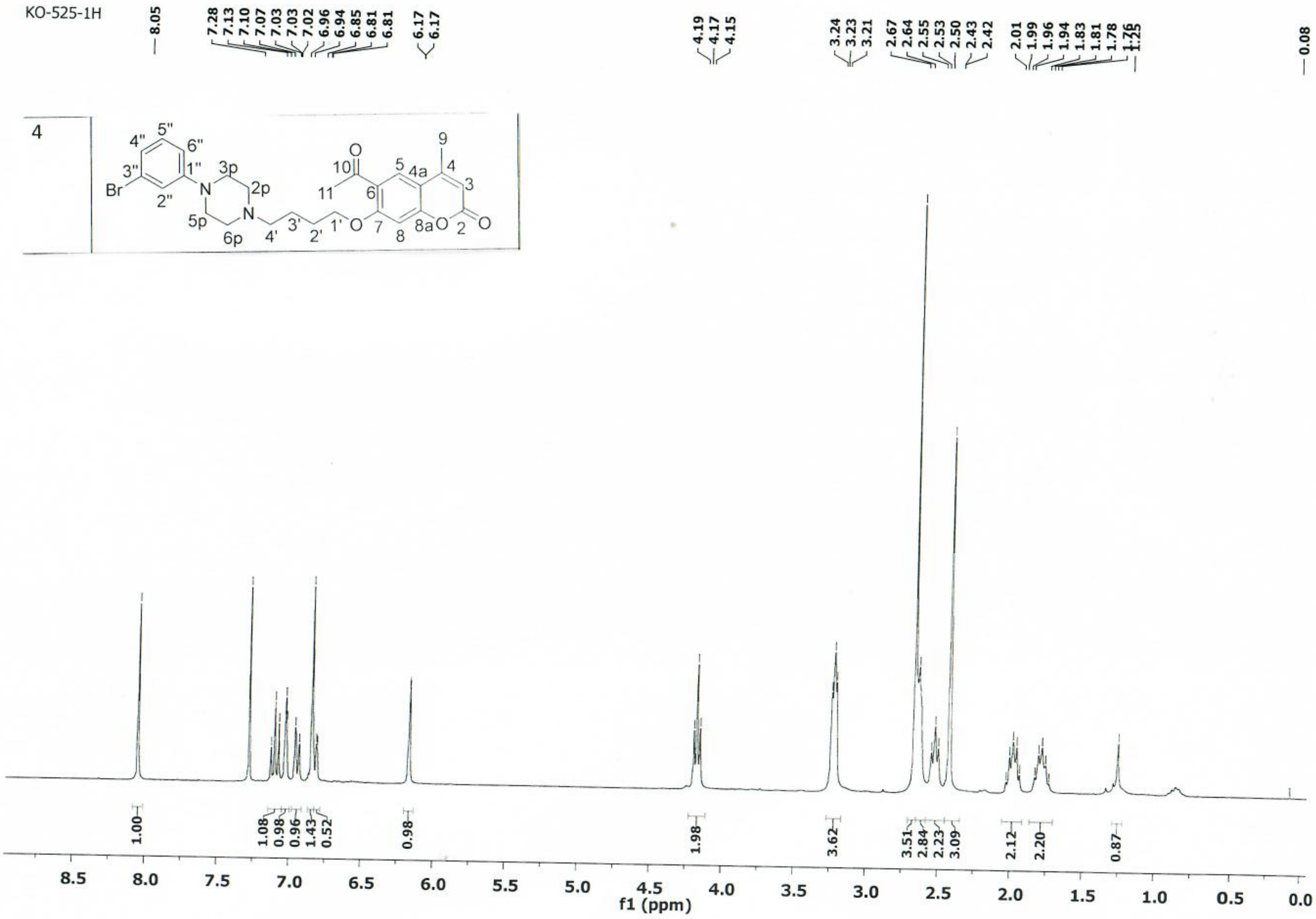

KO-525-1H
8.05
7.28 7.13 7.10 7.07 7.03 7.03 7.02 6.96 6.94 6.85 6.81 6.81
6.17 6.17
4.19 4.17 4.15
3.24 3.23 3.21
2.67 2.64 2.55 2.53 2.50 2.43 2.42
2.01 1.99 1.96 1.94 1.83 1.81 1.78 1.76 1.25
0.08
4
Br
1.00
1.08 0.98 0.96 1.43 0.52
0.98
1.98
3.62
3.51 2.84 2.23 3.09
2.12 2.20
0.87
8.5 8.0 7.5 7.0 6.5 6.0 5.5 5.0 4.5 4.0 3.5 3.0 2.5 2.0 1.5 1.0 0.5 0.0
f1 (ppm)

KO-525-13C

4

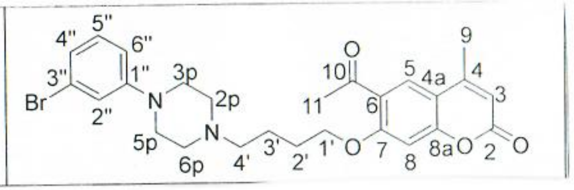

5''
4''
6''
3''
1''
3p
2p
Br
2''
N
N
5p
6p
4'
3'
2'
1'
O
7
8
8a
O
2
O
10
O
11
6
5
4a
4
9
3

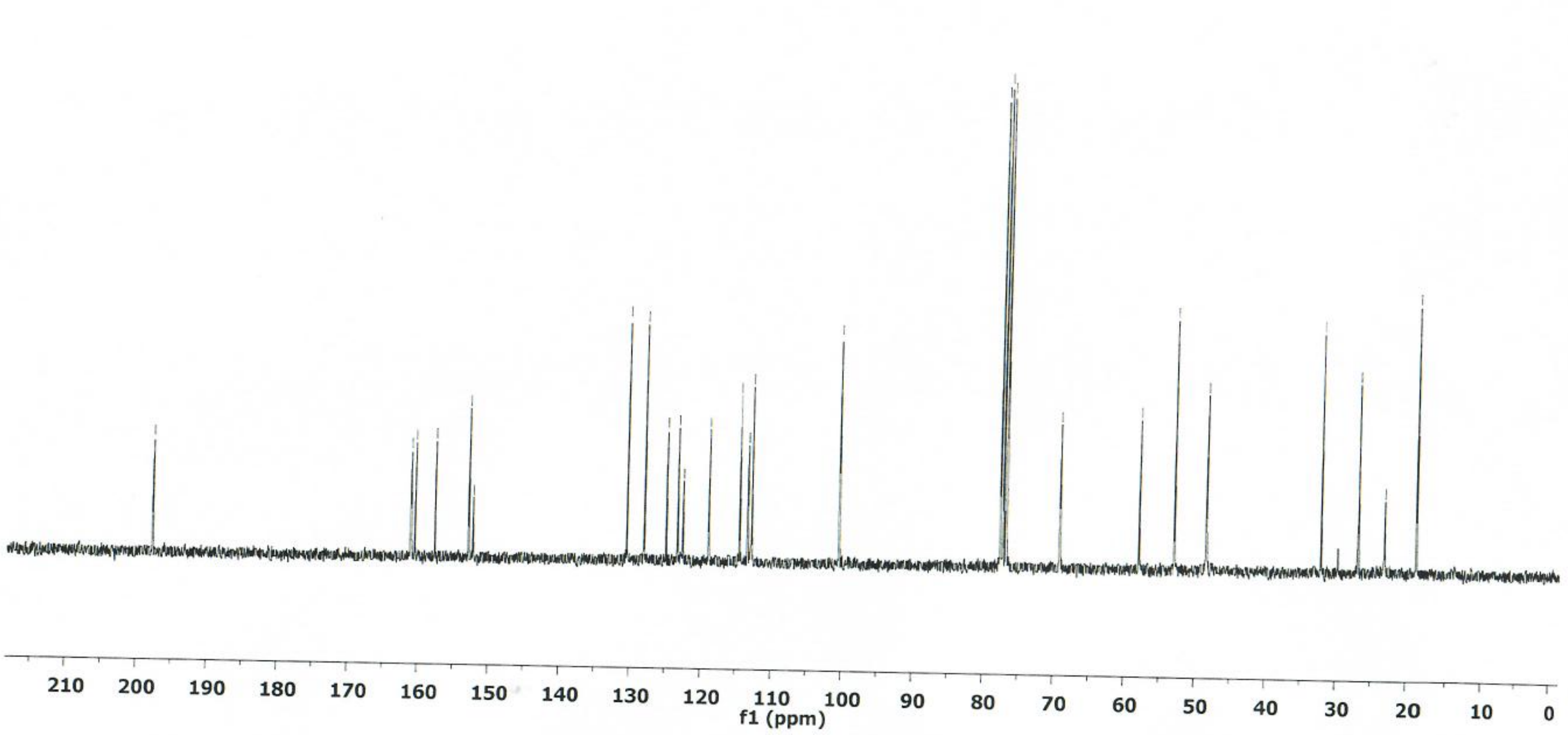

197.77
161.19
160.51
157.69
152.97
152.32
130.53
128.10
125.00
123.39
122.65
118.98
114.62
113.49
112.93
100.53
77.65
77.23
76.81
69.30
57.97
53.00
48.46
32.30
27.01
23.25
18.89
210
200
190
180
170
160
150
140
130
120
110
100
90
80
70
60
50
40
30
20
10
0
f1 (ppm)

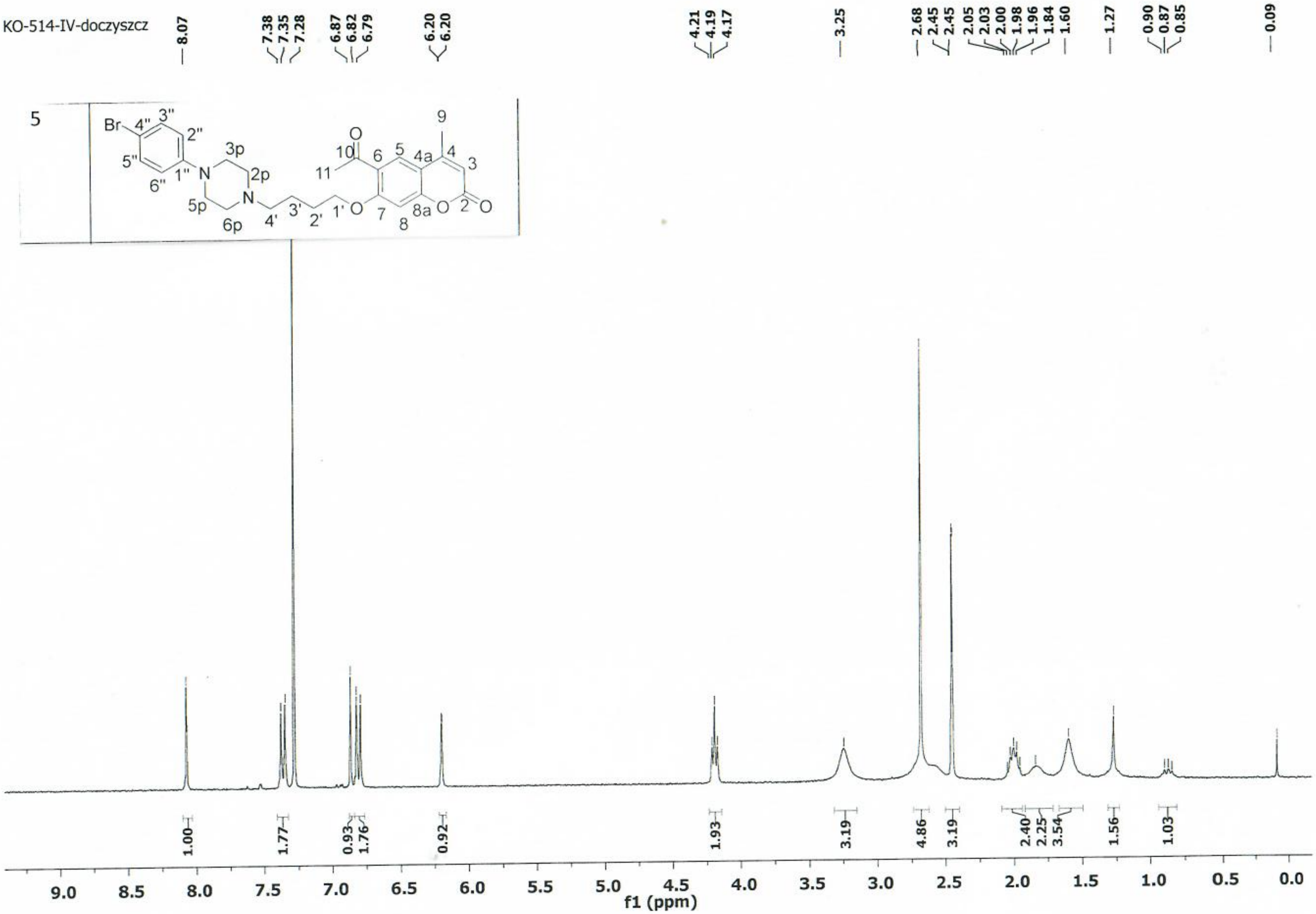
KO-514-IV-doczyszcz
8.07
7.38
7.35
7.28
6.87
6.82
6.79
6.20
6.20
4.21
4.19
4.17
3.25
2.68
2.45
2.45
2.05
2.03
2.00
1.98
1.96
1.84
1.60
1.27
0.90
0.87
0.85
0.09
5
Br
4''
3''
2''
5''
6''
1''
N
3p
2p
5p
6p
N
4'
3'
2'
1'
O
7
8
8a
O
2
O
3
4
4a
5
6
9
10
O
11
1.00
1.77
0.93
1.76
0.92
1.93
3.19
4.86
3.19
2.40
2.25
3.54
1.56
1.03
9.0
8.5
8.0
7.5
7.0
6.5
6.0
5.5
5.0
4.5
4.0
3.5
3.0
2.5
2.0
1.5
1.0
0.5
0.0
f1 (ppm)

KO-514-IV-13C

5

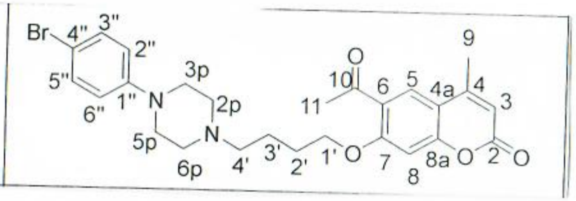

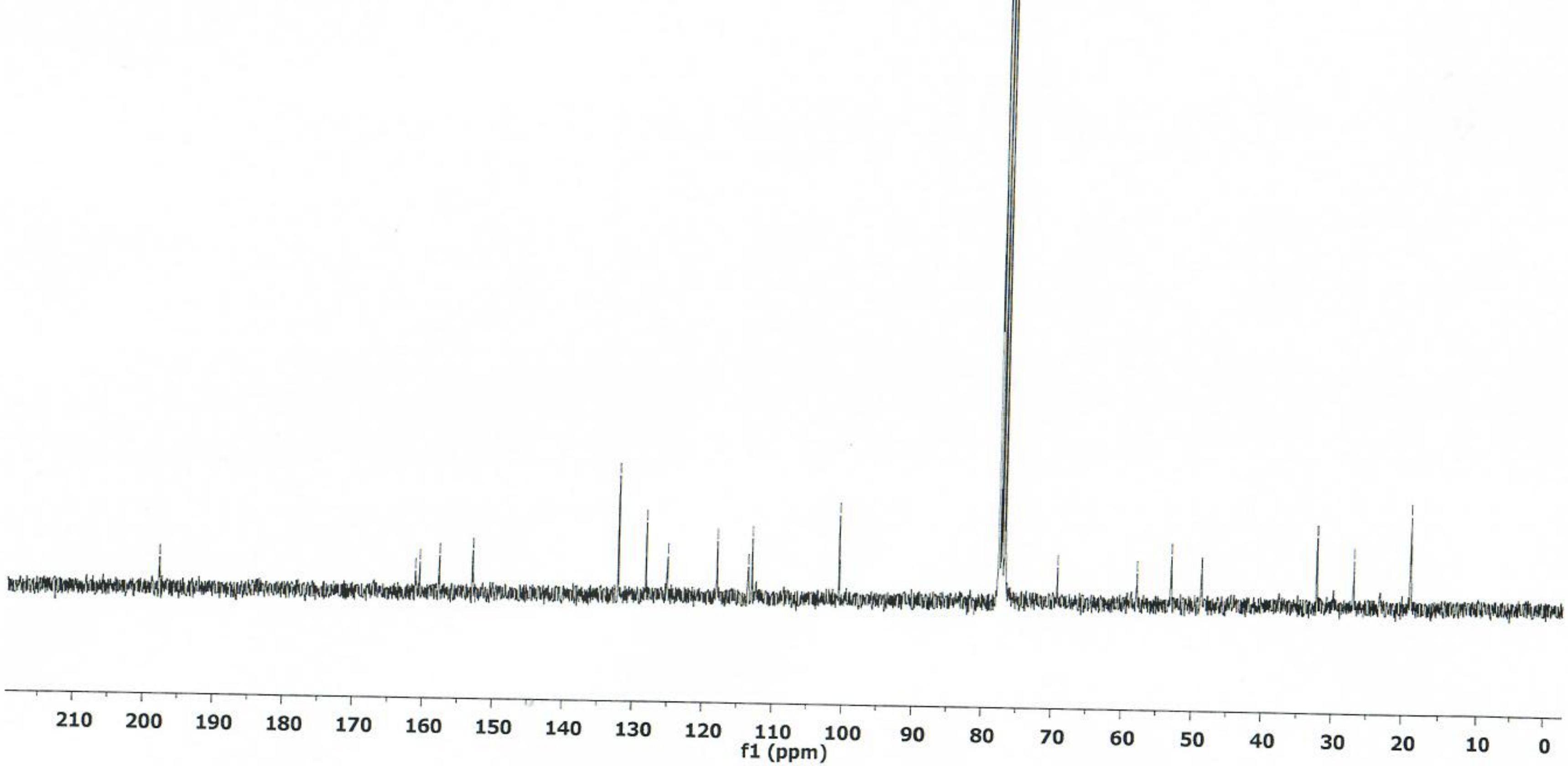

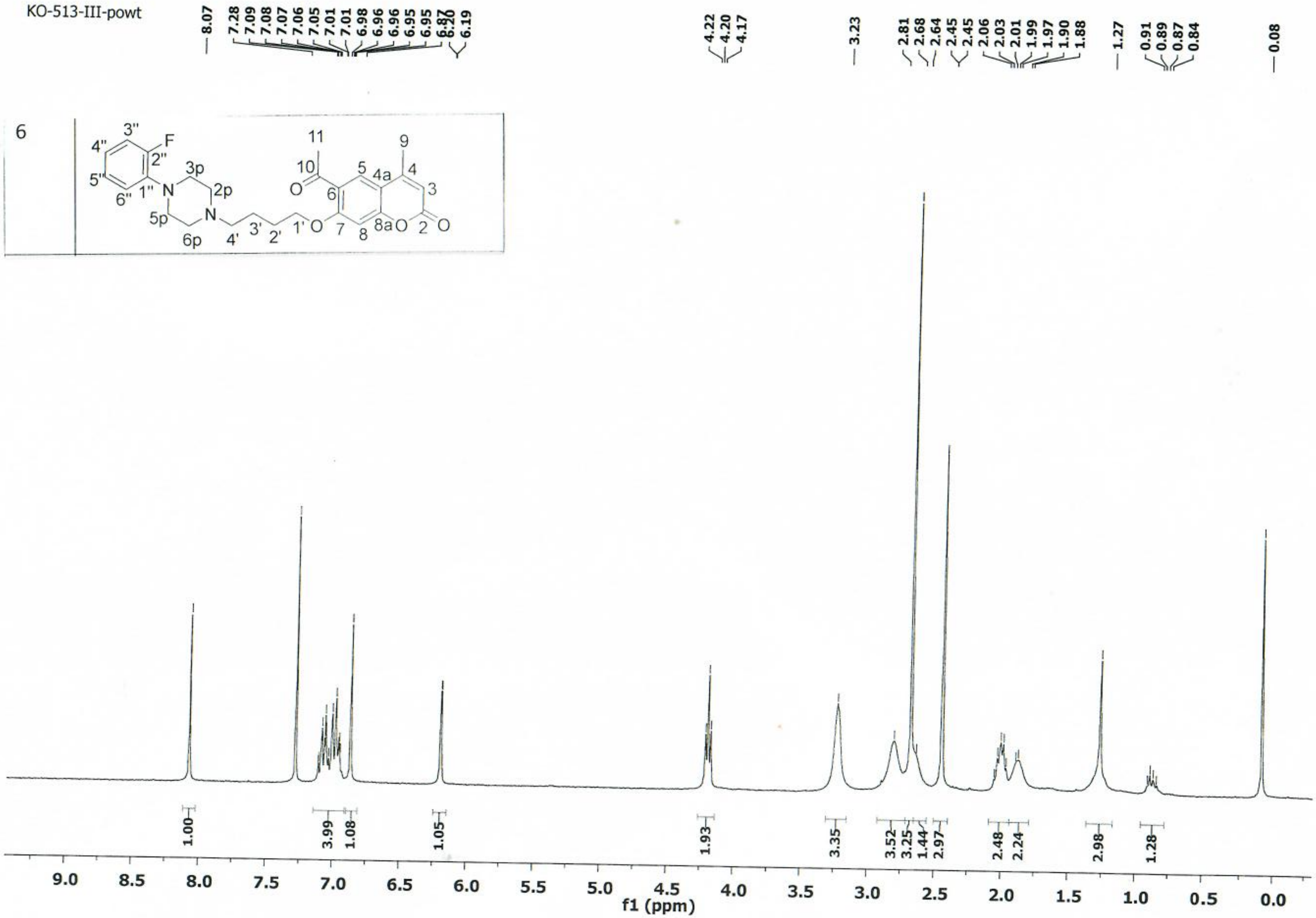
KO-513-III-powt
8.07
7.28
7.09
7.08
7.07
7.06
7.05
7.01
7.01
6.98
6.96
6.96
6.95
6.95
6.19
4.22
4.20
4.17
3.23
2.81
2.68
2.64
2.45
2.45
2.06
2.03
2.01
1.99
1.97
1.90
1.88
1.27
0.91
0.89
0.87
0.84
0.08
6
F
11
9
10
5
4a
4
3
O
6
7
8
8a
O
2
O
3"
4"
2"
5"
6"
1"
N
3p
2p
5p
6p
N
4'
3'
2'
1'
1.00
3.99
1.08
1.05
1.93
3.35
3.52
3.25
1.44
2.97
2.48
2.24
2.98
1.28
f1 (ppm)

6

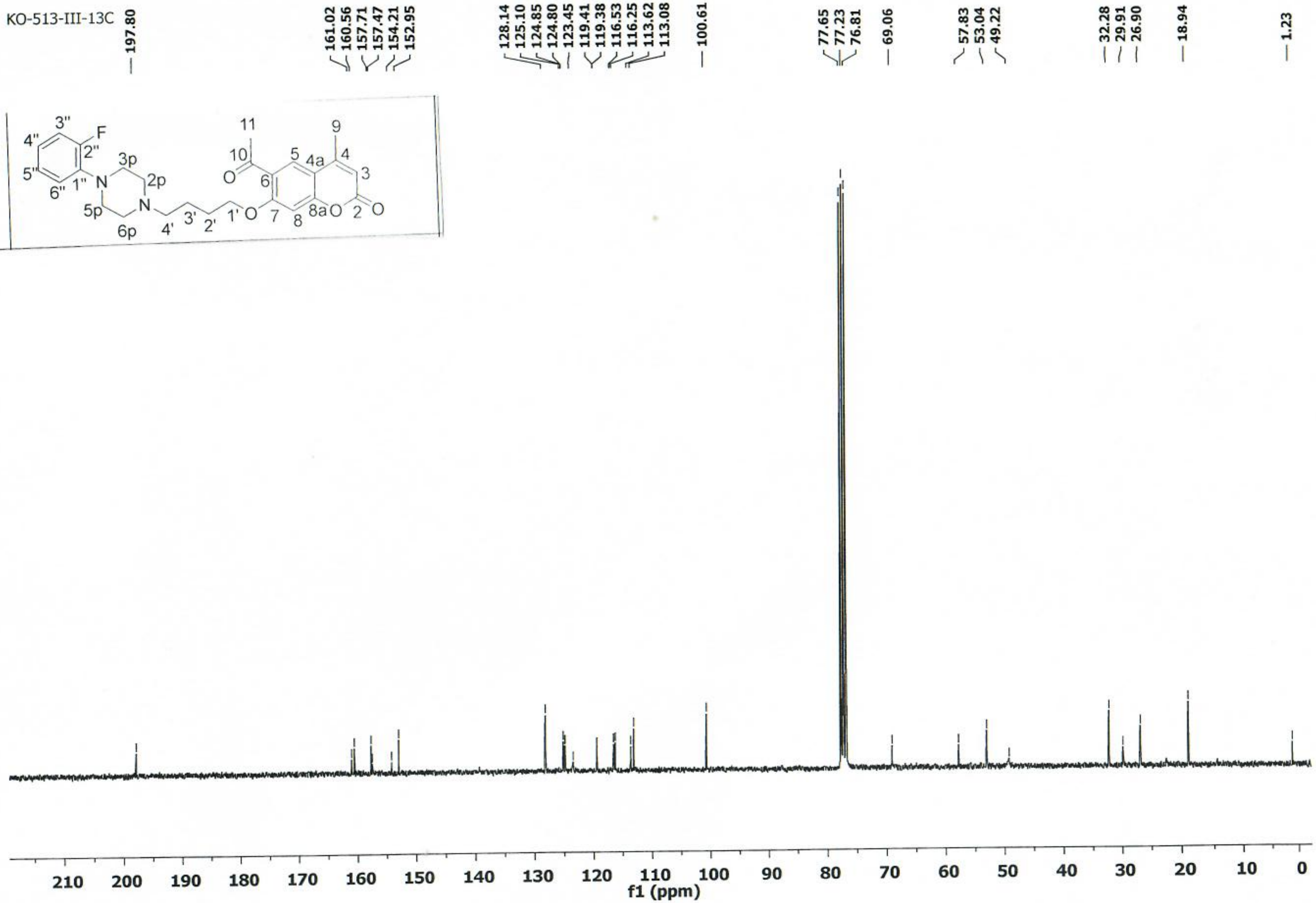
3''
F
4''
2''
3p
5''
1''
N
2p
6''
5p
N
6p
4'
3'
2'
1'
O
7
8
8a
O
2
O
11
10
O
6
5
4a
9
4
3
KO-513-III-13C
197.80
161.02
160.56
157.71
157.47
154.21
152.95
128.14
125.10
124.85
124.80
123.45
119.41
119.38
116.53
116.25
113.62
113.08
100.61
77.65
77.23
76.81
69.06
57.83
53.04
49.22
32.28
29.91
26.90
18.94
1.23
210
200
190
180
170
160
150
140
130
120
110
100
90
80
70
60
50
40
30
20
10
0
f1 (ppm)

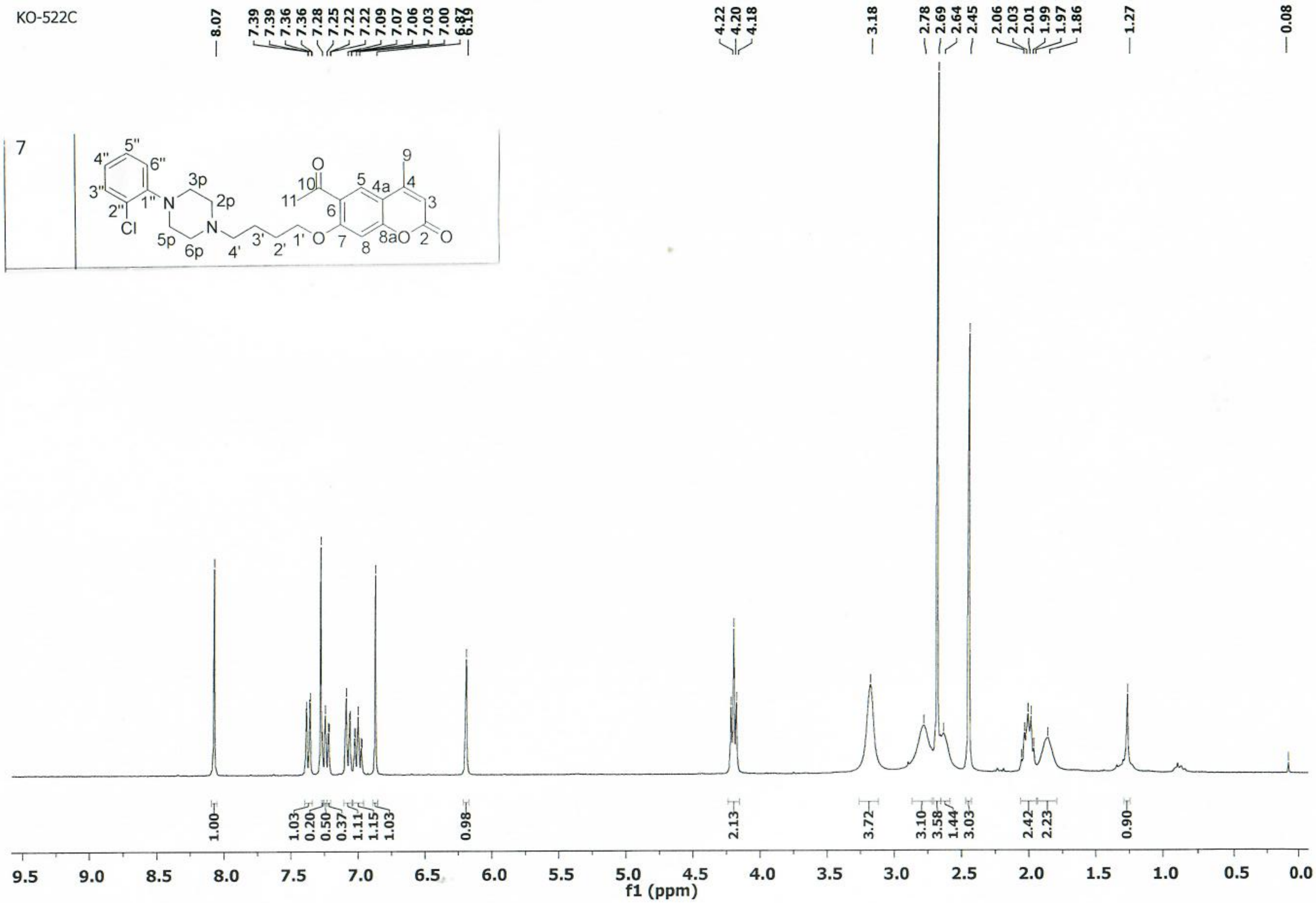
KO-522C
8.07
7.39 7.39 7.36 7.36 7.28 7.25 7.22 7.22 7.09 7.07 7.06 7.03 7.00 6.87 6.19
4.22 4.20 4.18
3.18
2.78 2.69 2.64 2.45
2.06 2.03 2.01 1.99 1.97 1.86
1.27
0.08
7
1.00 1.03 0.20 0.50 0.37 1.11 1.15 1.03 0.98 2.13 3.72 3.10 3.58 1.44 3.03 2.42 2.23 0.90
9.5 9.0 8.5 8.0 7.5 7.0 6.5 6.0 5.5 5.0 4.5 4.0 3.5 3.0 2.5 2.0 1.5 1.0 0.5 0.0
f1 (ppm)

7

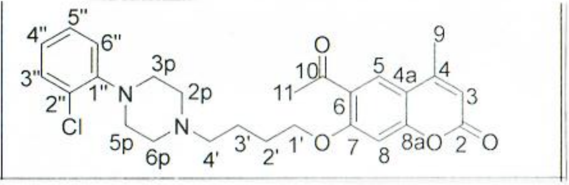

5''
4''
6''
3''
2''
1''
Cl
N
N
3p
2p
5p
6p
4'
3'
2'
1'
O
10
11
6
5
4a
9
4
3
7
8
8a
O
2
O

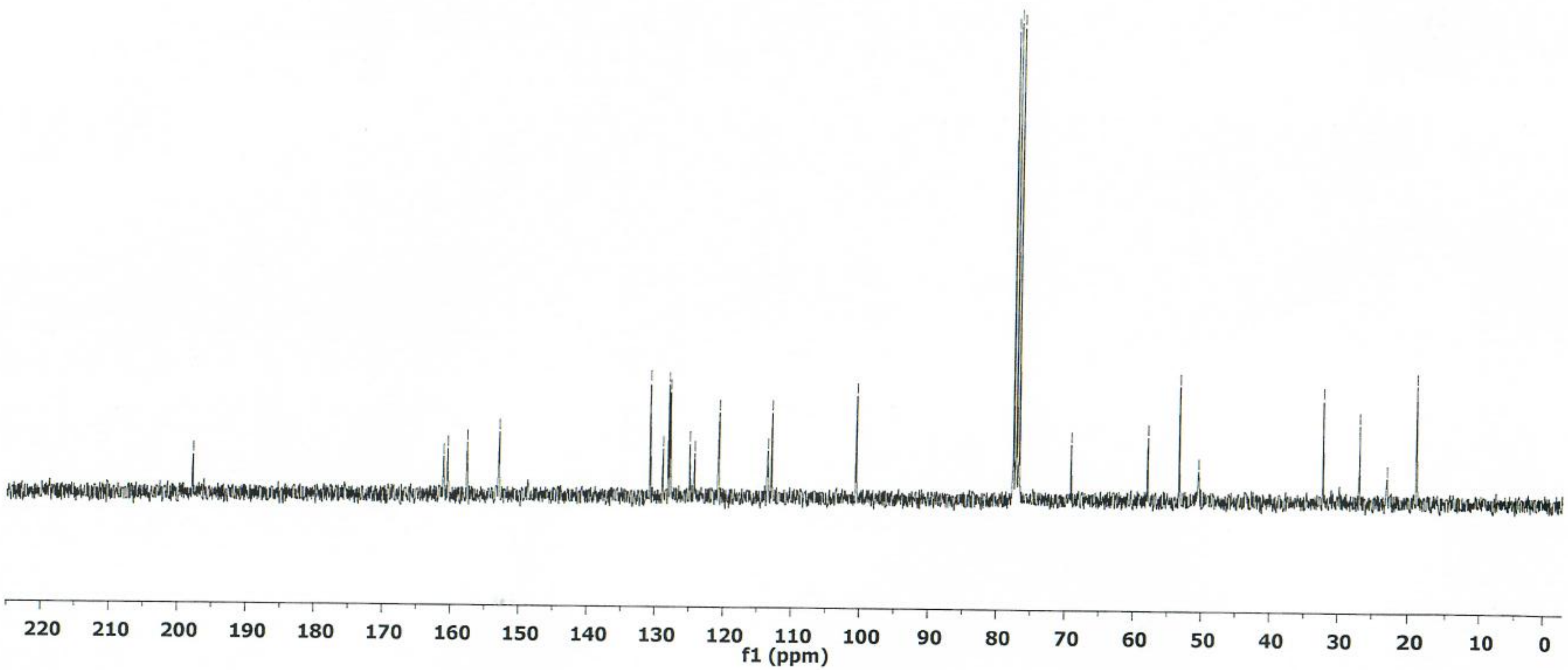

KO-522-C-13C
197.83
161.16
160.57
157.73
152.99
130.82
128.92
128.15
127.91
125.05
124.33
120.73
113.56
113.00
100.58
77.65
77.23
76.81
69.24
58.01
53.35
50.52
32.33
27.01
22.95
18.93
220 210 200 190 180 170 160 150 140 130 120 110 100 90 80 70 60 50 40 30 20 10 0
f1 (ppm)

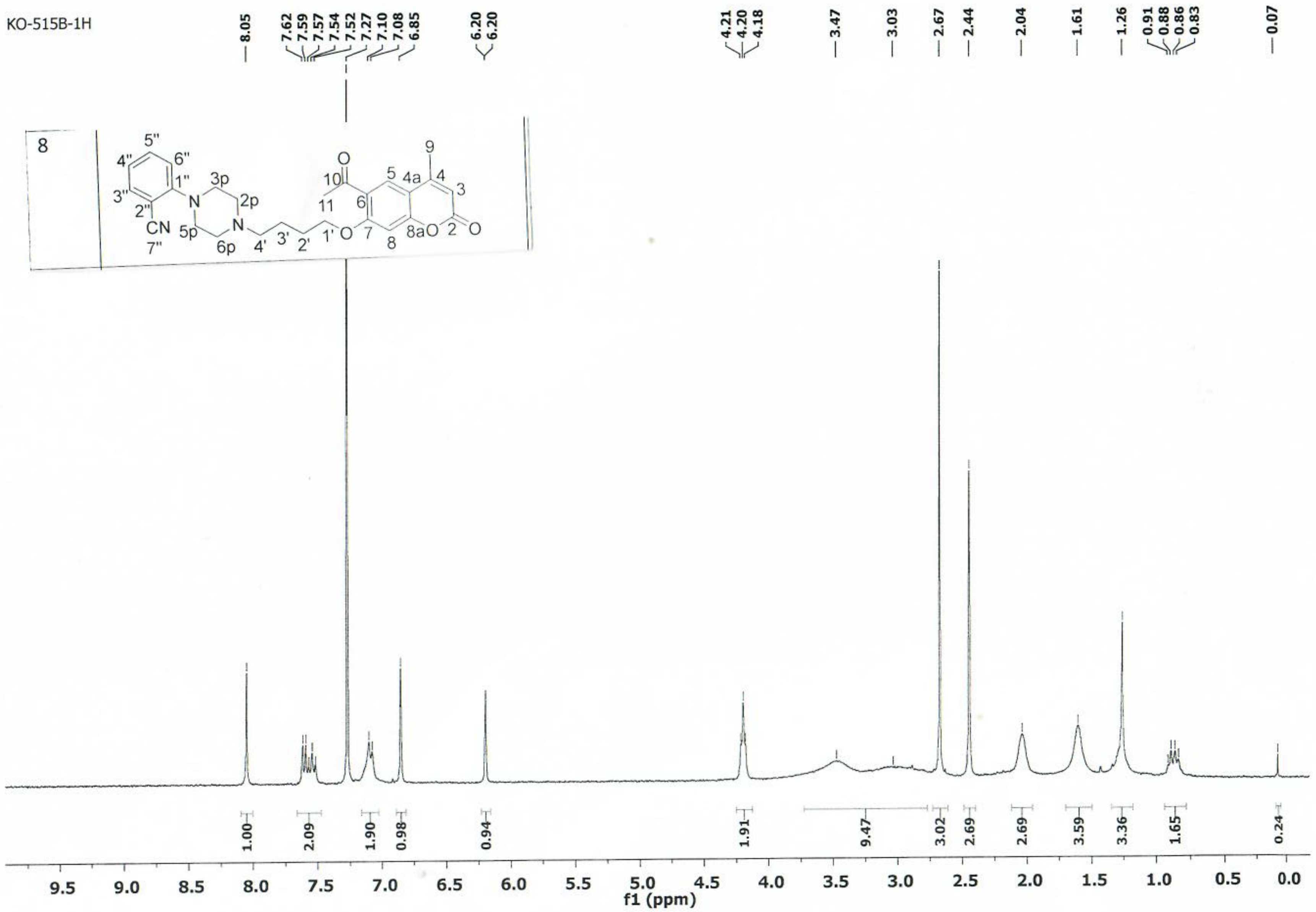

KO-515B-1H
8.05
7.62
7.59
7.57
7.54
7.52
7.27
7.10
7.08
6.85
6.20
6.20
4.21
4.20
4.18
3.47
3.03
2.67
2.44
2.04
1.61
1.26
0.91
0.88
0.86
0.83
0.07
8
1.00
2.09
1.90
0.98
0.94
1.91
9.47
3.02
2.69
2.69
3.59
3.36
1.65
0.24
9.5
9.0
8.5
8.0
7.5
7.0
6.5
6.0
5.5
5.0
4.5
4.0
3.5
3.0
2.5
2.0
1.5
1.0
0.5
0.0
f1 (ppm)

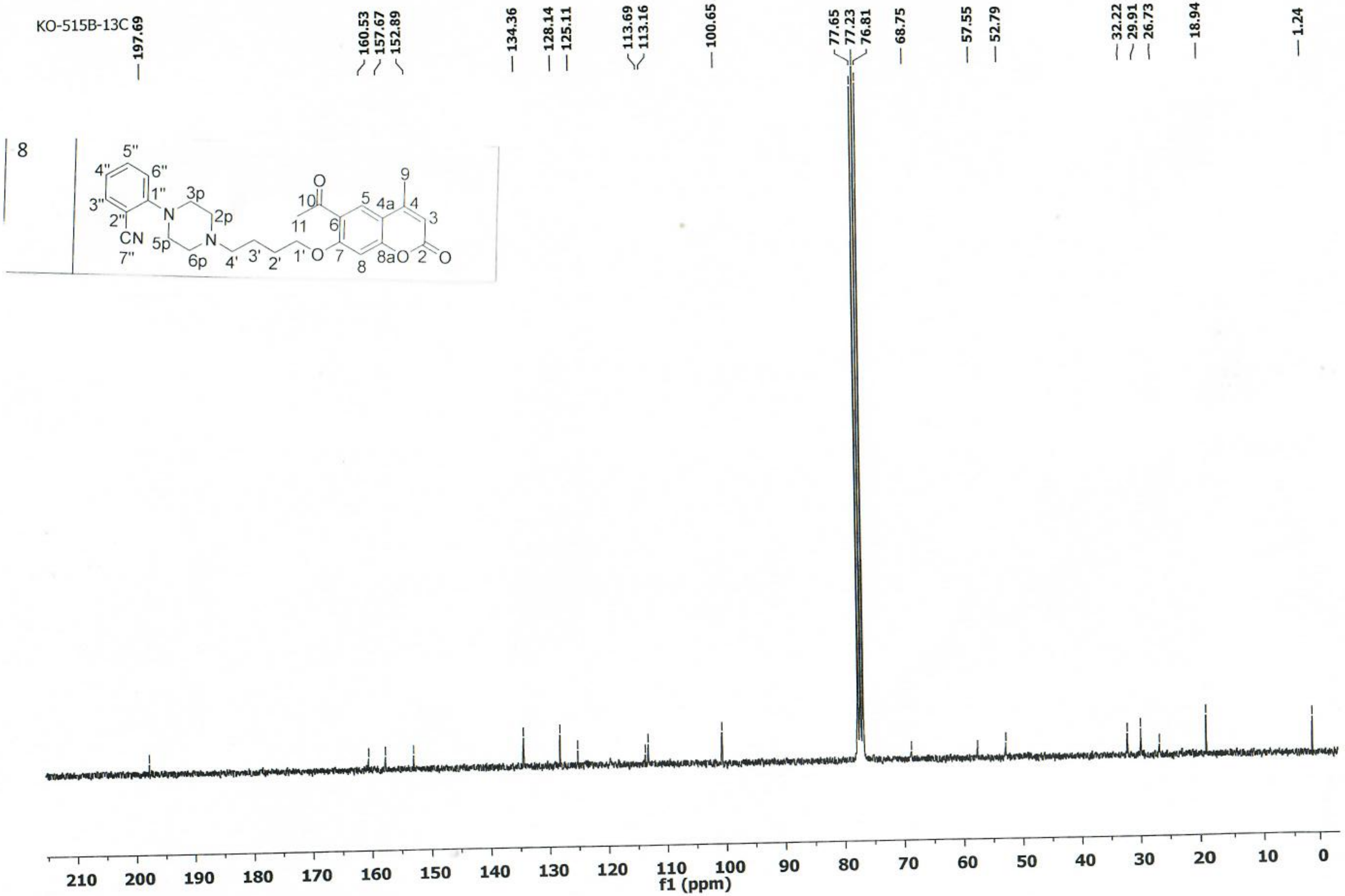
KO-515B-13C
197.69
160.53
157.67
152.89
134.36
128.14
125.11
113.69
113.16
100.65
77.65
77.23
76.81
68.75
57.55
52.79
32.22
29.91
26.73
18.94
1.24
8
5''
4''
6''
3''
1''
2''
CN
7''
3p
2p
5p
6p
N
N
4'
3'
2'
1'
O
11
10
6
5
4a
4
9
3
7
8
8a
O
2
210
200
190
180
170
160
150
140
130
120
110
100
90
80
70
60
50
40
30
20
10
0
f1 (ppm)

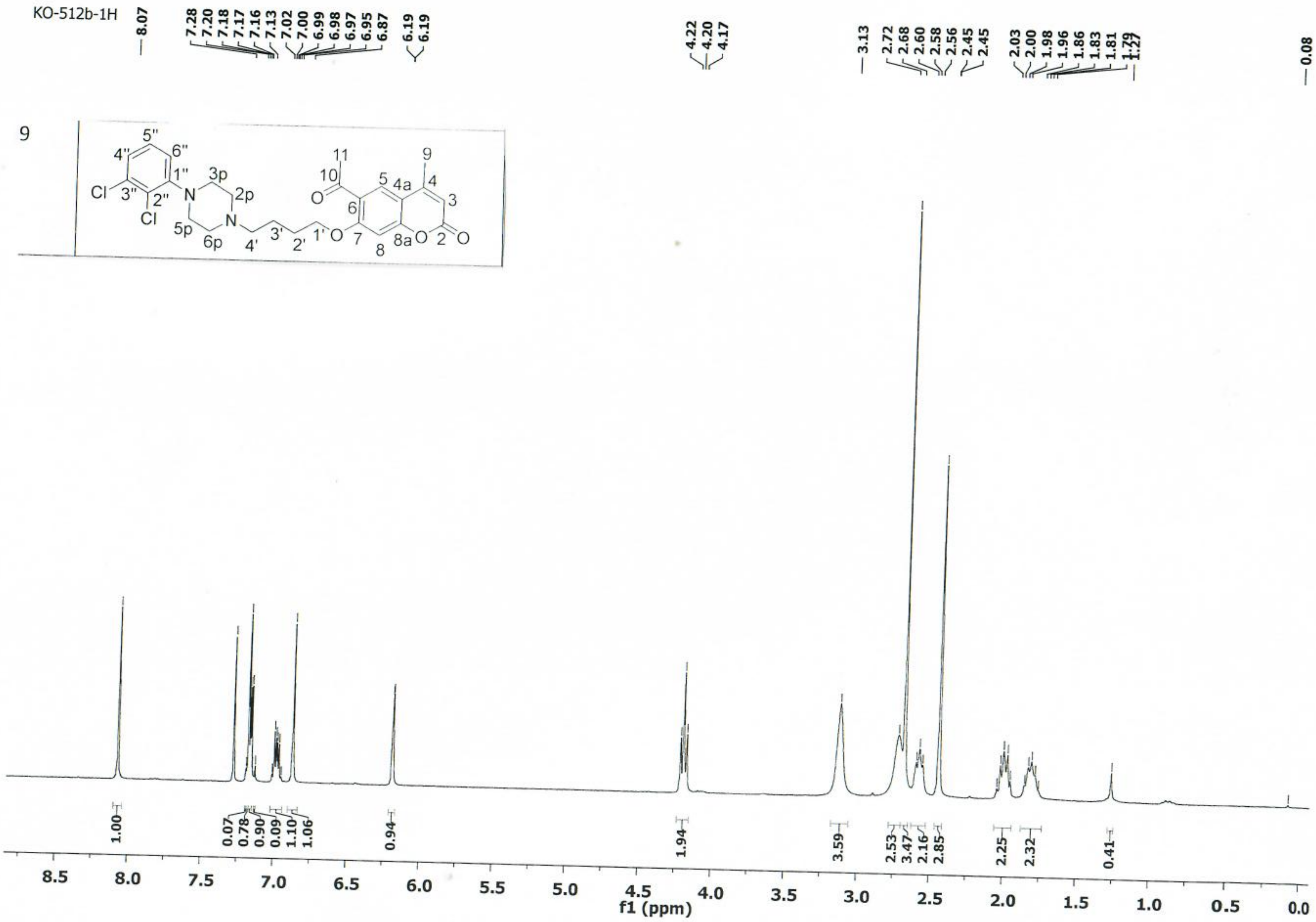

KO-512b-1H
8.07
7.28 7.20 7.18 7.17 7.16 7.13 7.02 7.00 6.99 6.98 6.97 6.95 6.87
6.19 6.19
4.22 4.20 4.17
3.13
2.72 2.68 2.60 2.58 2.56 2.45 2.45
2.03 2.00 1.98 1.96 1.86 1.83 1.81 1.79 1.27
0.08
9
1.00
0.07 0.78 0.90 0.09 1.10 1.06
0.94
1.94
3.59
2.53 3.47 2.16 2.85
2.25 2.32
0.41
8.5 8.0 7.5 7.0 6.5 6.0 5.5 5.0 4.5 4.0 3.5 3.0 2.5 2.0 1.5 1.0 0.5 0.0
f1 (ppm)

KO-512b-13C

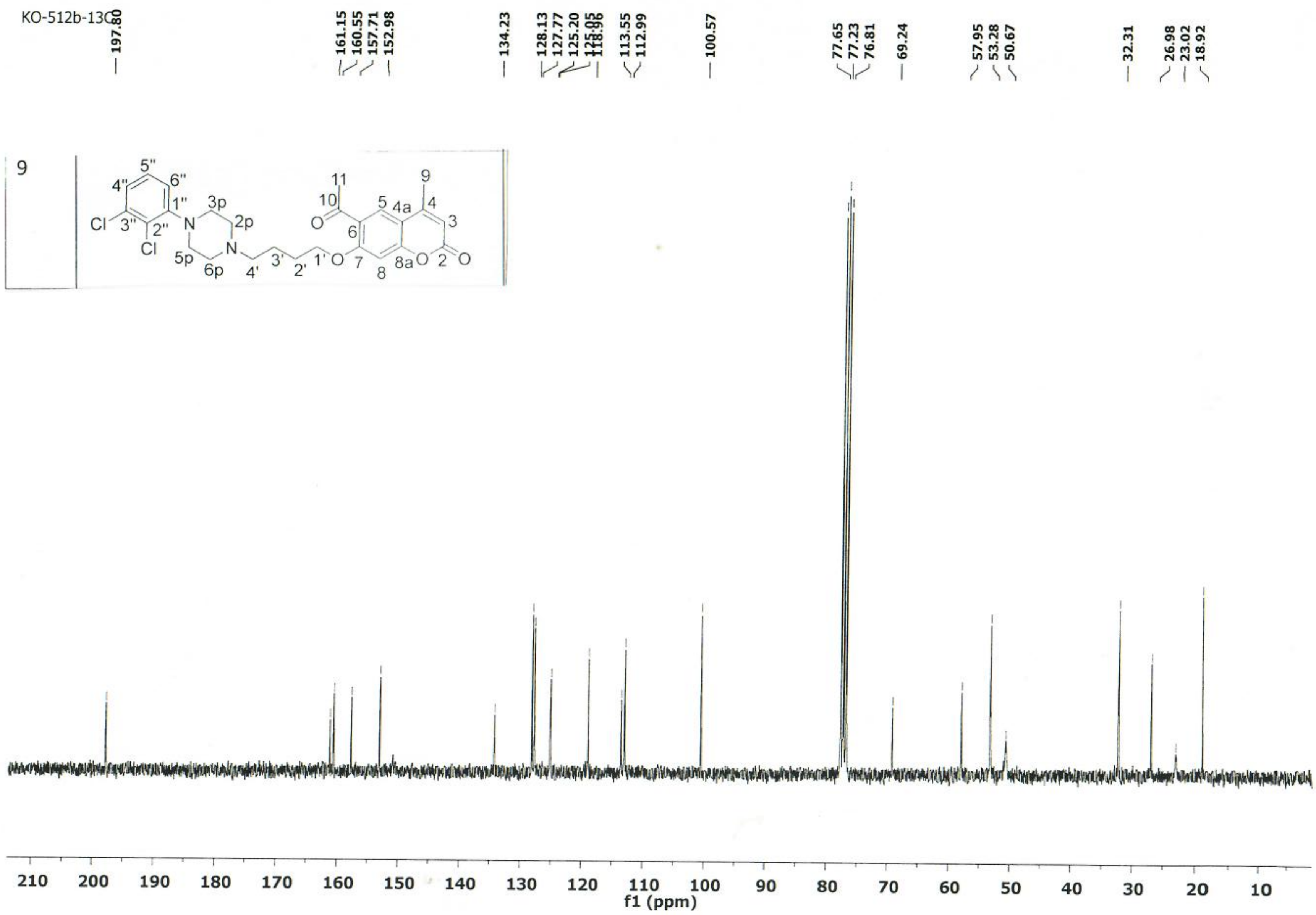
197.80
161.15
160.55
157.71
152.98
134.23
128.13
127.77
125.20
113.55
112.99
100.57
77.65
77.23
76.81
69.24
57.95
53.28
50.67
32.31
26.98
23.02
18.92
9
210 200 190 180 170 160 150 140 130 120 110 100 90 80 70 60 50 40 30 20 10
f1 (ppm)

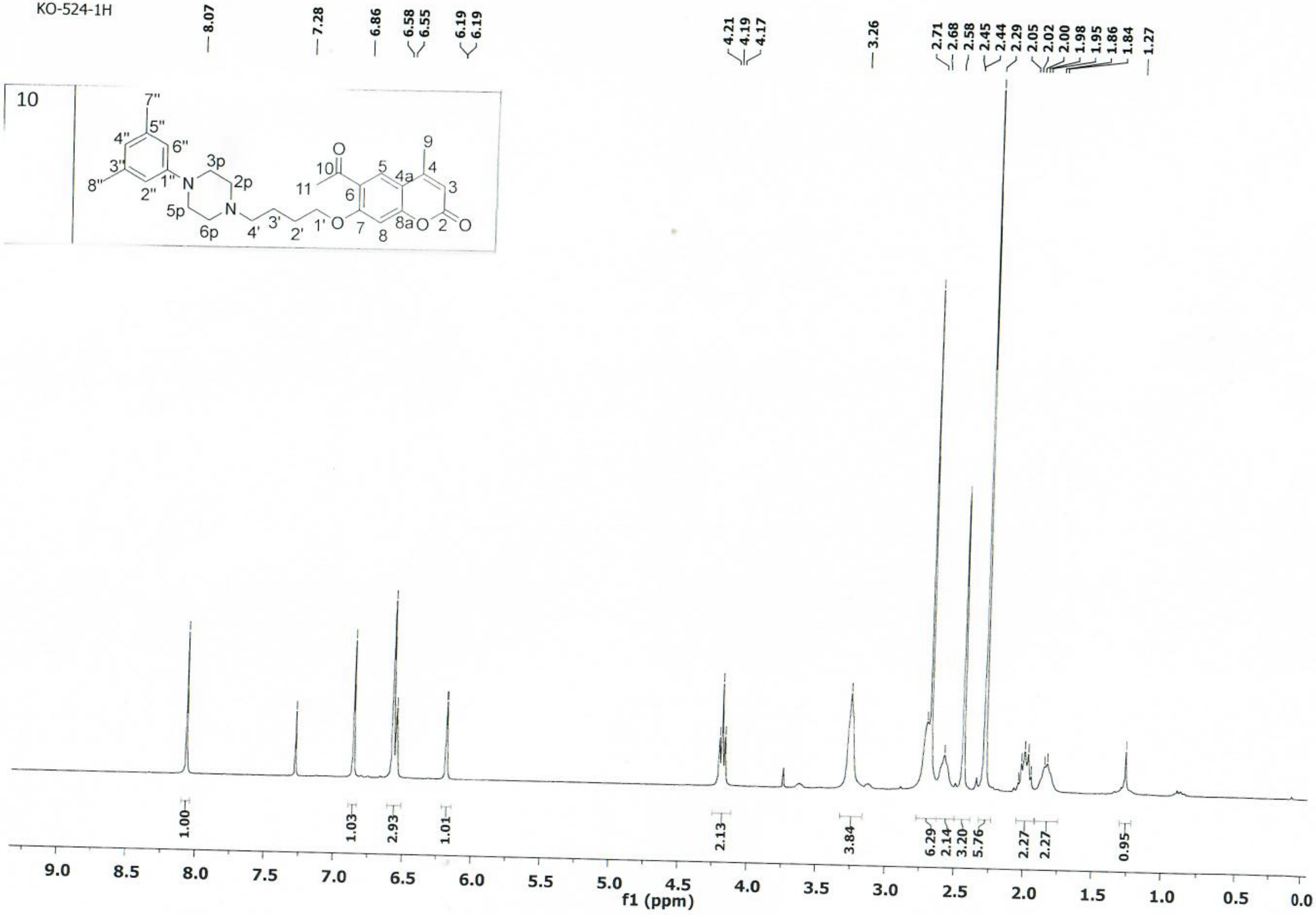

KO-524-1H
8.07
7.28
6.86
6.58
6.55
6.19
6.19
4.21
4.19
4.17
3.26
2.71
2.68
2.58
2.45
2.44
2.29
2.05
2.02
2.00
1.98
1.95
1.86
1.84
1.27
10
1.00
1.03
2.93
1.01
2.13
3.84
6.29
2.14
3.20
5.76
2.27
2.27
0.95
9.0 8.5 8.0 7.5 7.0 6.5 6.0 5.5 5.0 4.5 4.0 3.5 3.0 2.5 2.0 1.5 1.0 0.5 0.0
f1 (ppm)

KO-524-13C

10

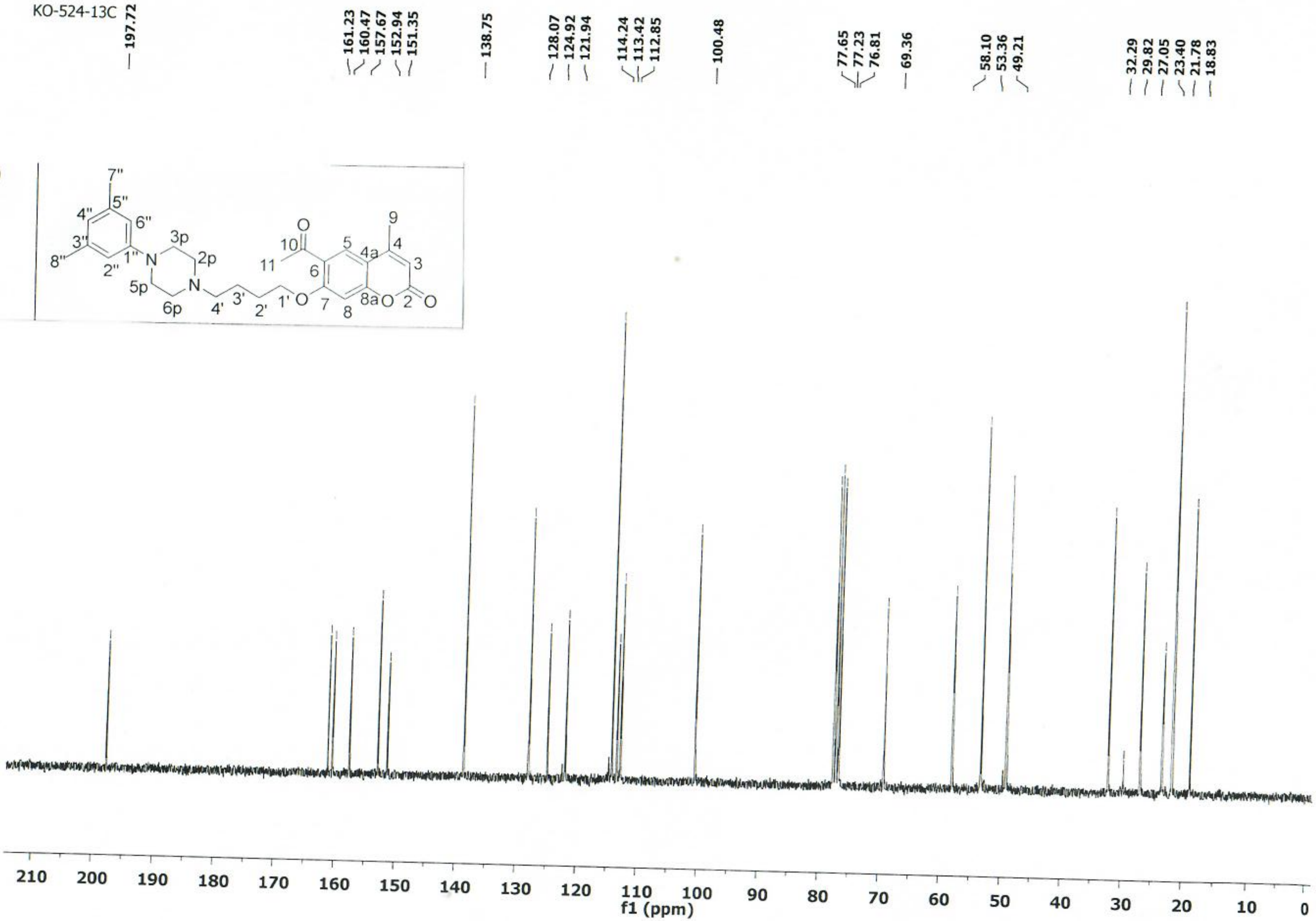
197.72
161.23
160.47
157.67
152.94
151.35
138.75
128.07
124.92
121.94
114.24
113.42
112.85
100.48
77.65
77.23
76.81
69.36
58.10
53.36
49.21
32.29
29.82
27.05
23.40
21.78
18.83
7''
5''
4''
6''
3''
8''
1''
2''
N
3p
2p
5p
6p
N
4'
3'
2'
1'
O
10
O
11
6
7
5
4a
9
4
3
8
8a
O
2
O
210 200 190 180 170 160 150 140 130 120 110 100 90 80 70 60 50 40 30 20 10 0
f1 (ppm)

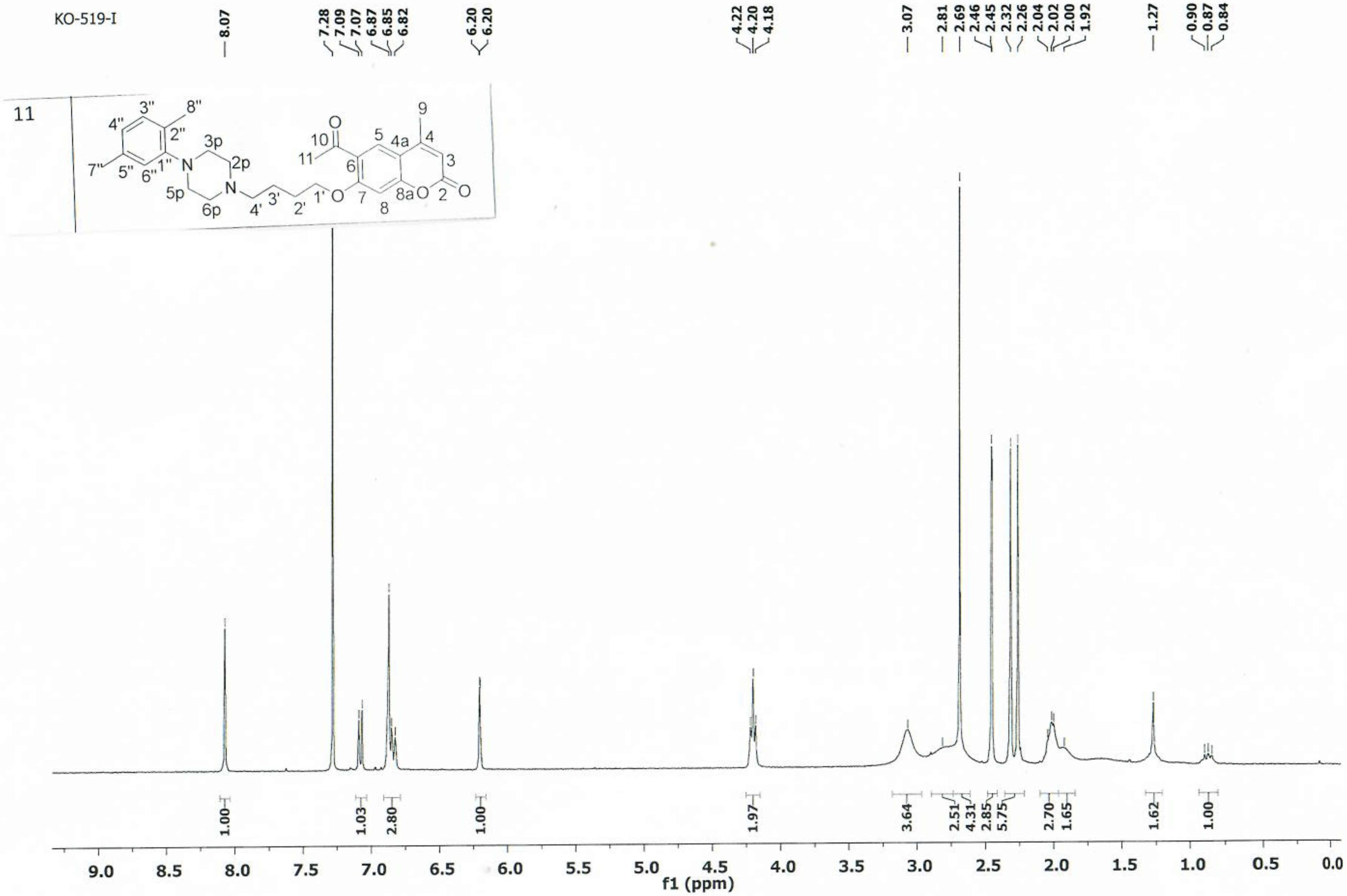

KO-519-I
11
8.07
7.28
7.09
7.07
6.87
6.85
6.82
6.20
6.20
4.22
4.20
4.18
3.07
2.81
2.69
2.46
2.45
2.32
2.26
2.04
2.02
2.00
1.92
1.27
0.90
0.87
0.84
1.00
1.03
2.80
1.00
1.97
3.64
2.51
4.31
2.85
5.75
2.70
1.65
1.62
1.00
9.0 8.5 8.0 7.5 7.0 6.5 6.0 5.5 5.0 4.5 4.0 3.5 3.0 2.5 2.0 1.5 1.0 0.5 0.0
f1 (ppm)

KO-519-I-13C

11

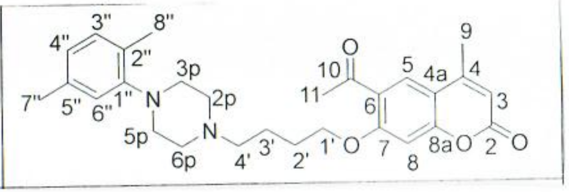

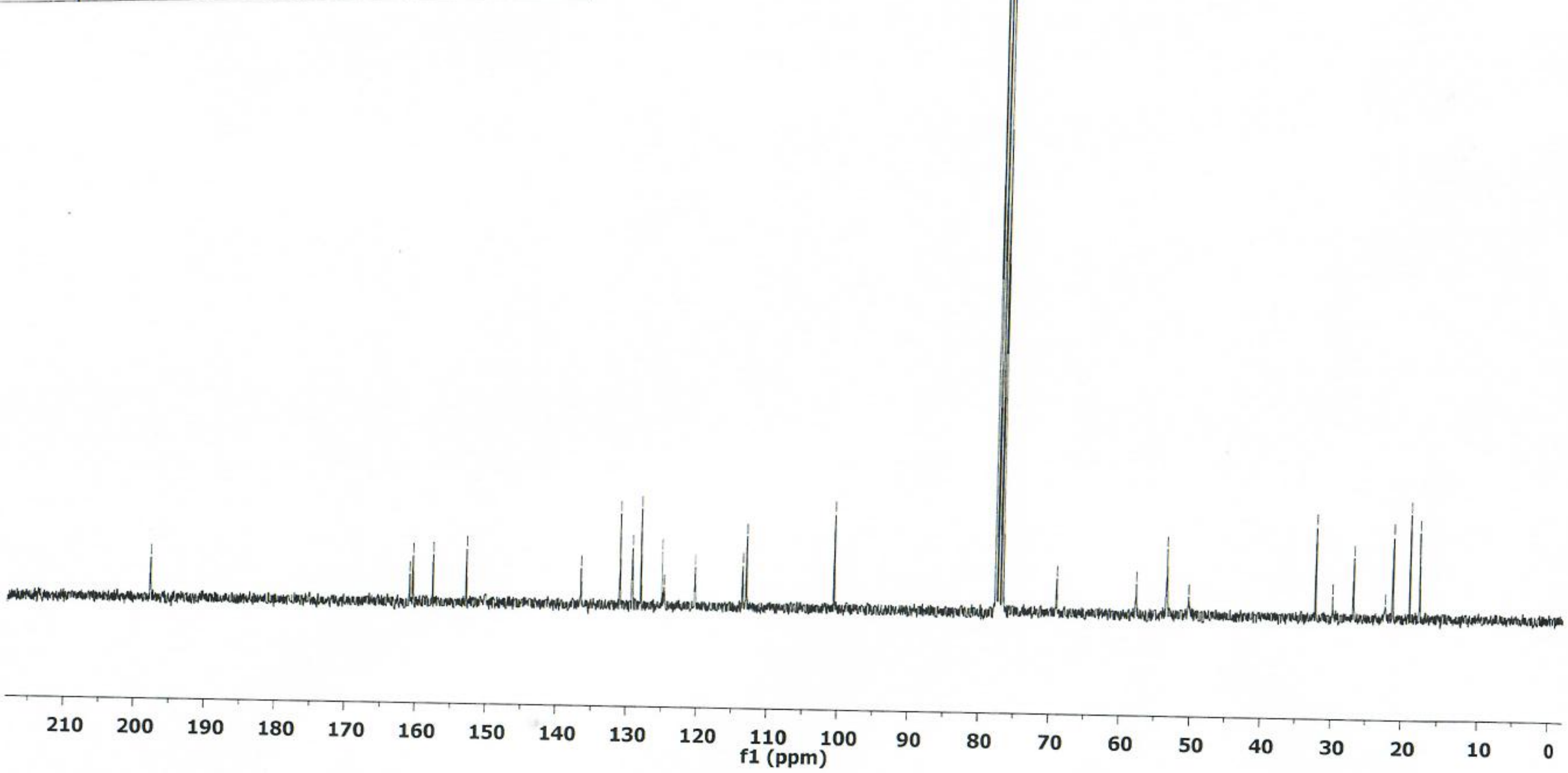

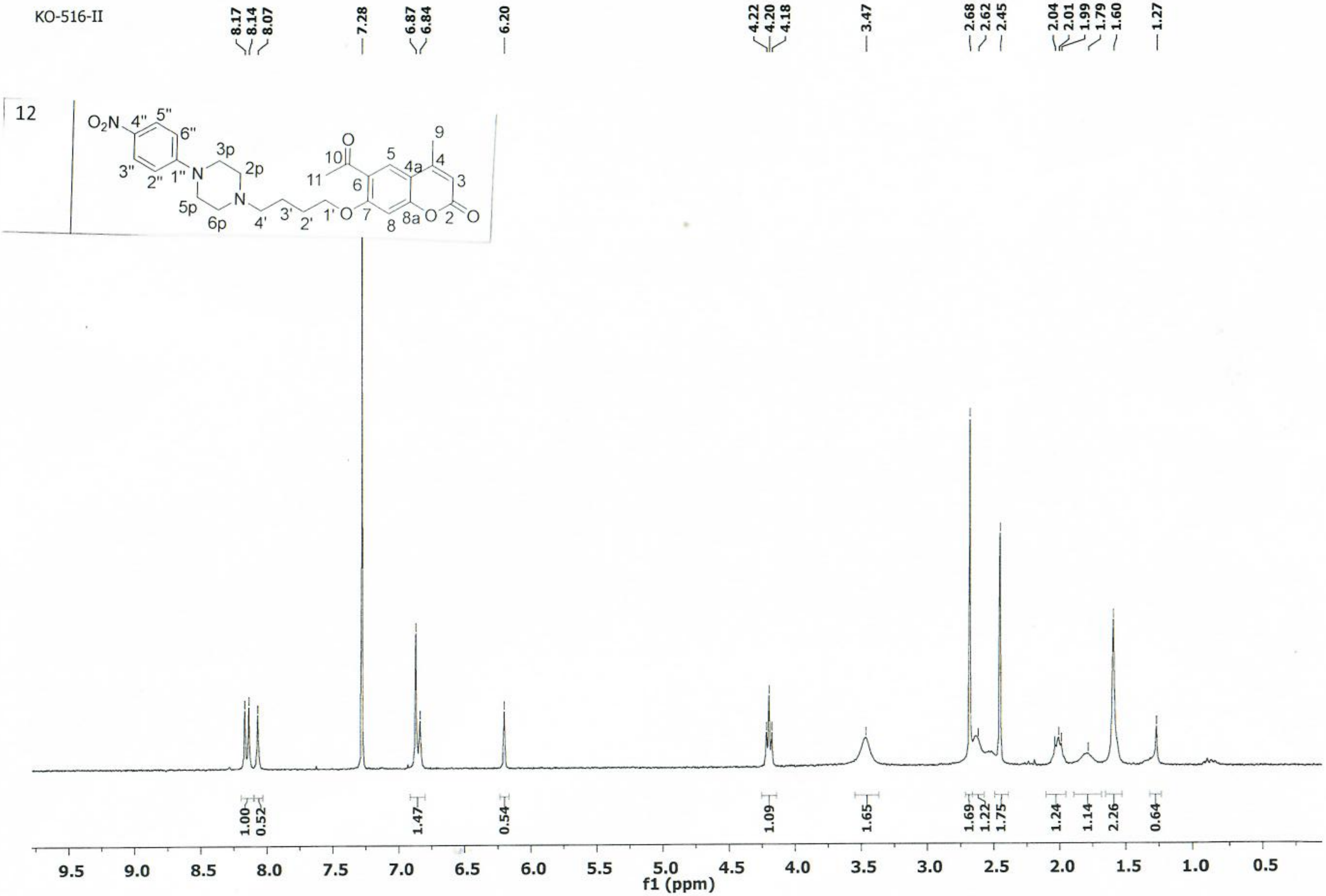
KO-516-II
8.17
8.14
8.07
7.28
6.87
6.84
6.20
4.22
4.20
4.18
3.47
2.68
2.62
2.45
2.04
2.01
1.99
1.79
1.60
1.27
12
1.00
0.52
1.47
0.54
1.09
1.65
1.69
1.22
1.75
1.24
1.14
2.26
0.64
9.5
9.0
8.5
8.0
7.5
7.0
6.5
6.0
5.5
5.0
4.5
4.0
3.5
3.0
2.5
2.0
1.5
1.0
0.5
f1 (ppm)

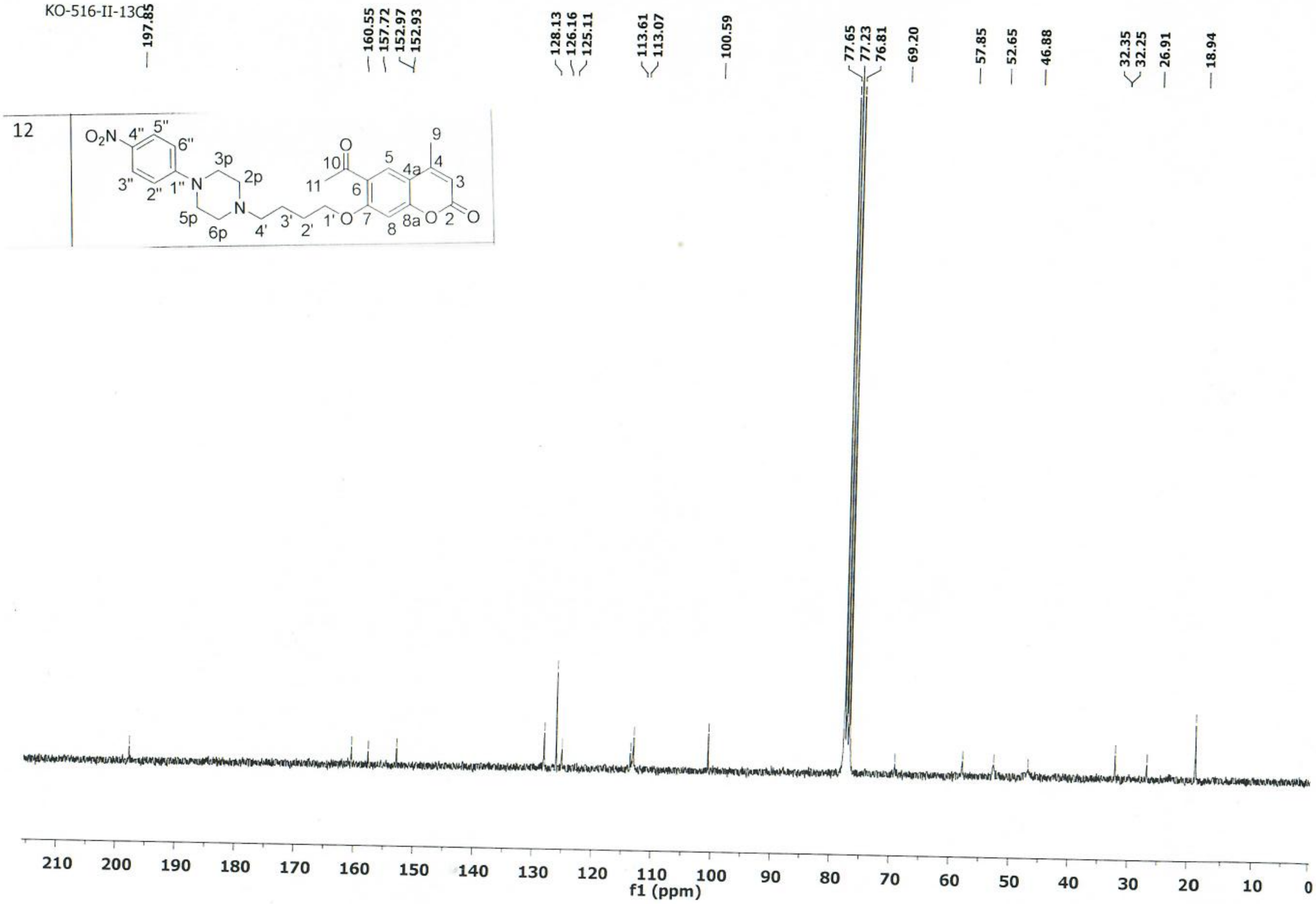
KO-516-II-13C
197.85
160.55
157.72
152.97
152.93
128.13
126.16
125.11
113.61
113.07
100.59
77.65
77.23
76.81
69.20
57.85
52.65
46.88
32.35
32.25
26.91
18.94
12
O_2N
210 200 190 180 170 160 150 140 130 120 110 100 90 80 70 60 50 40 30 20 10 0
f1 (ppm)

KO-520-III-1H

13

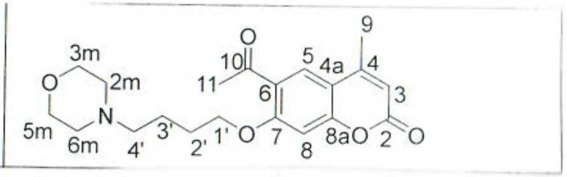

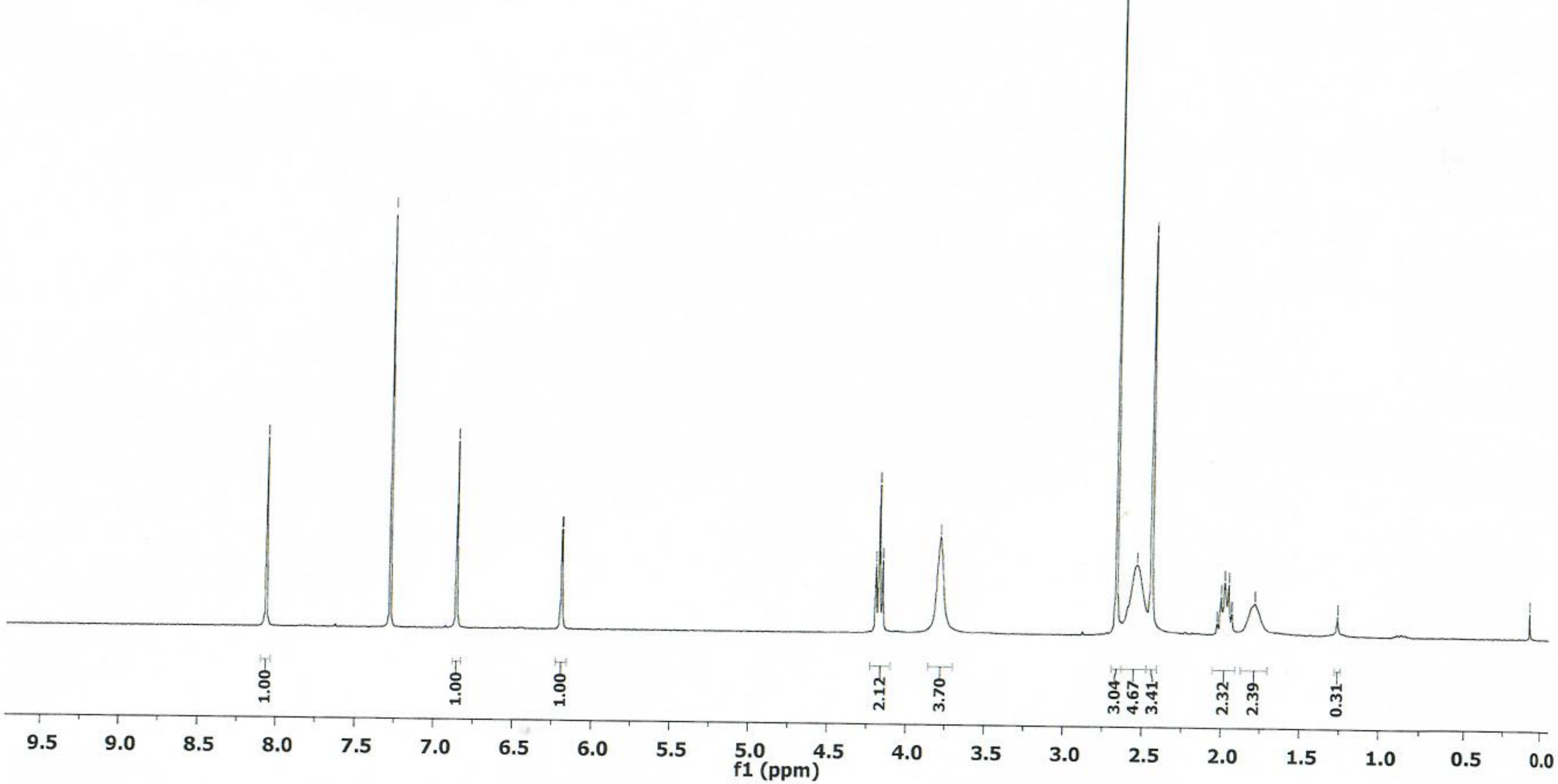

KO-520-III-13C

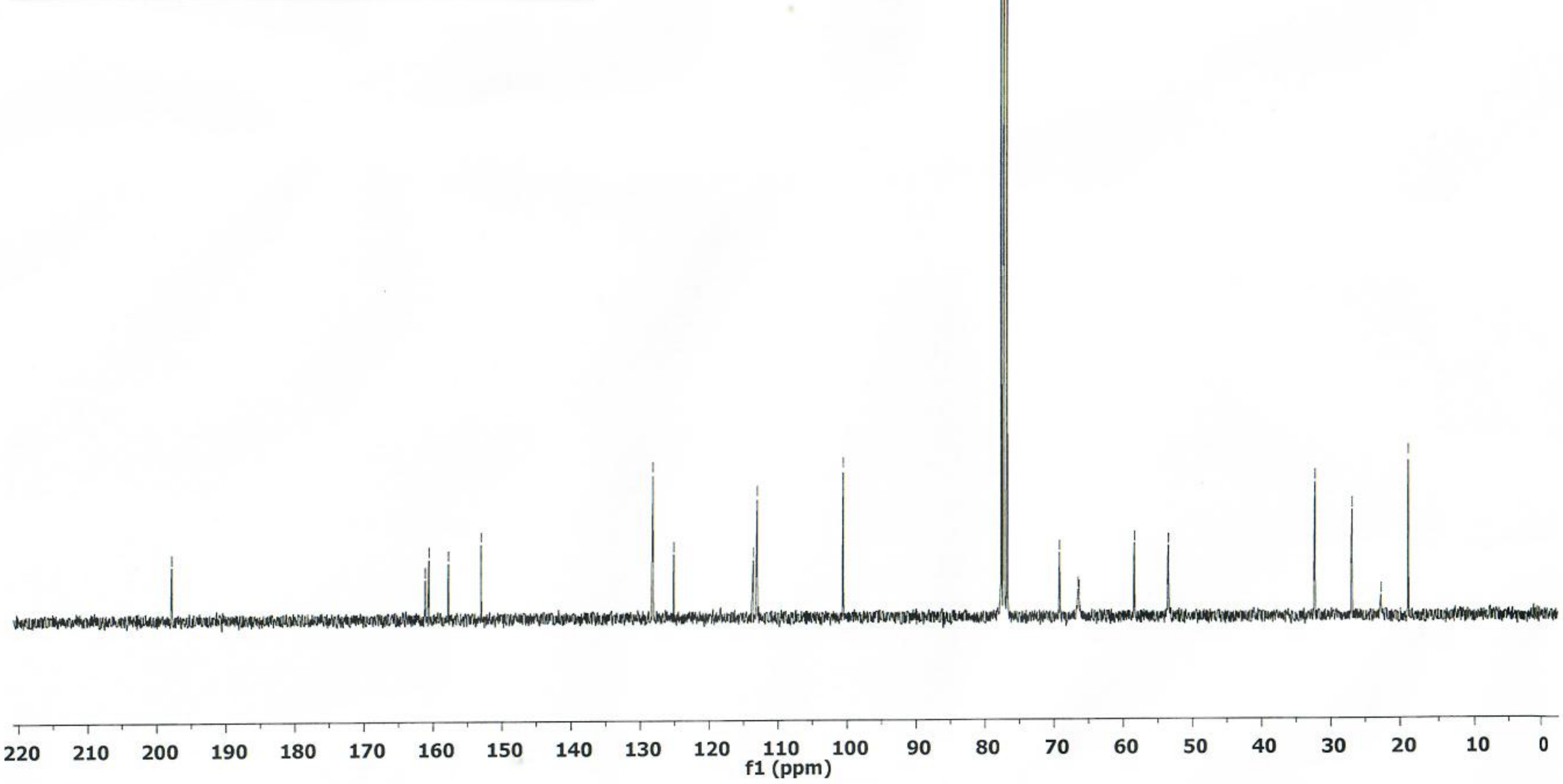

13

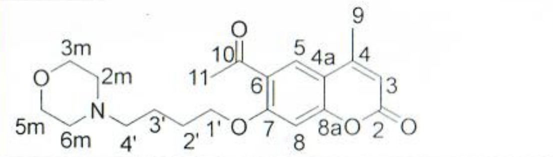

KO-517

14

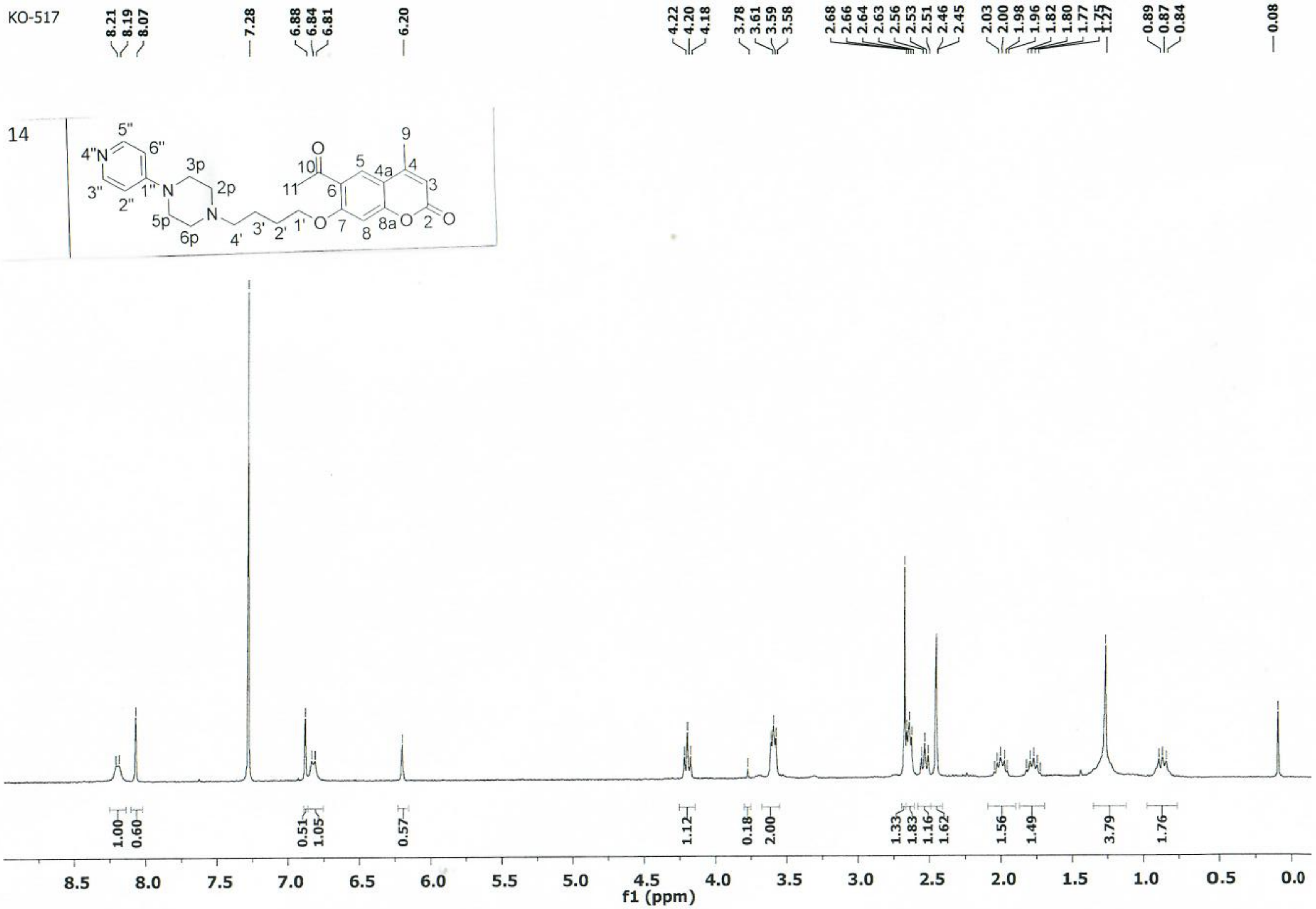
8.21
8.19
8.07
7.28
6.88
6.84
6.81
6.20
4.22
4.20
4.18
3.78
3.61
3.59
3.58
2.68
2.66
2.64
2.63
2.56
2.53
2.51
2.46
2.45
2.03
2.00
1.98
1.96
1.82
1.80
1.77
1.75
1.27
0.89
0.87
0.84
0.08
1.00
0.60
0.51
1.05
0.57
1.12
0.18
2.00
1.33
1.83
1.16
1.62
1.56
1.49
3.79
1.76
8.5
8.0
7.5
7.0
6.5
6.0
5.5
5.0
4.5
4.0
3.5
3.0
2.5
2.0
1.5
1.0
0.5
0.0
f1 (ppm)

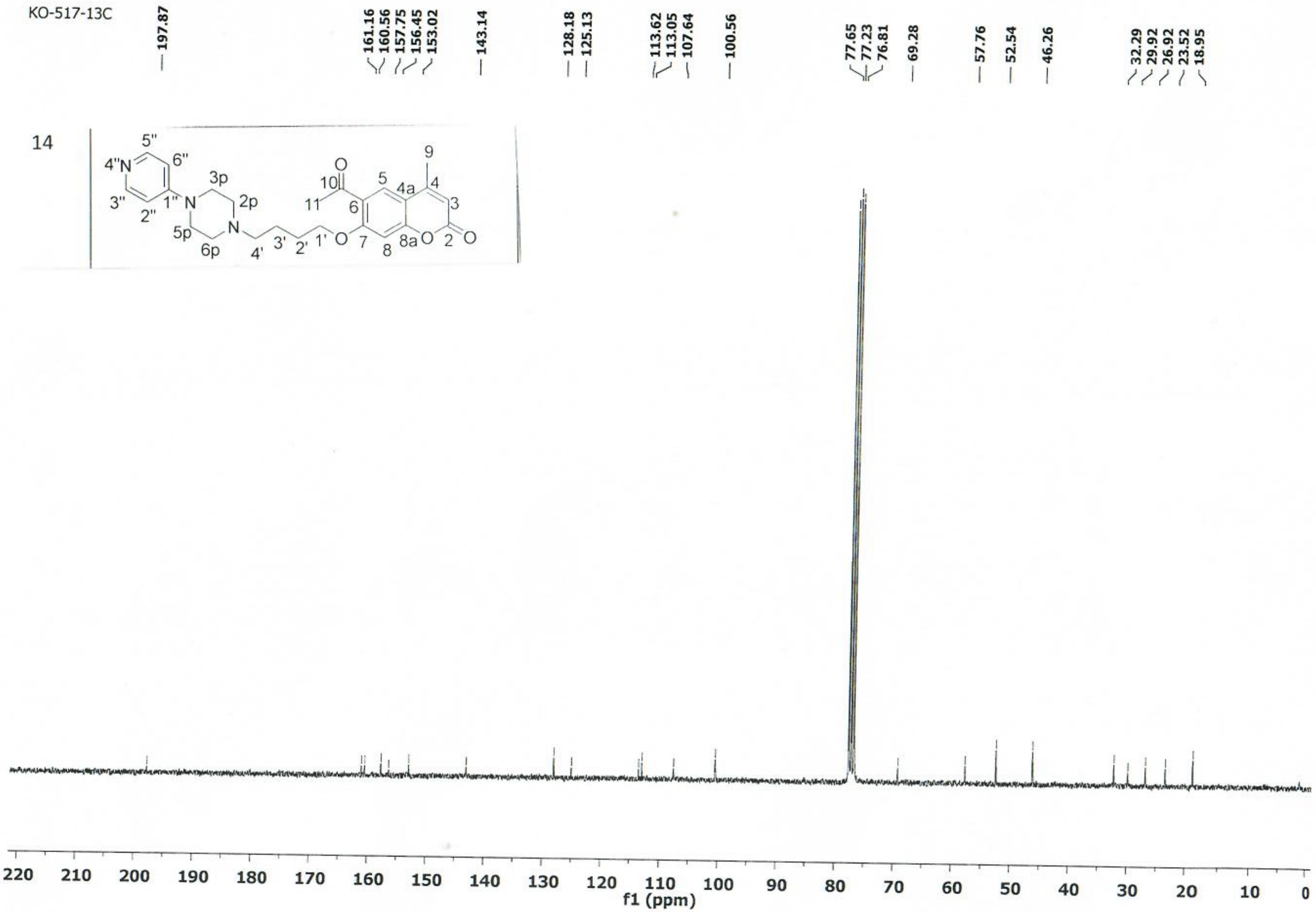
KO-517-13C
14
197.87
161.16
160.56
157.75
156.45
153.02
143.14
128.18
125.13
113.62
113.05
107.64
100.56
77.65
77.23
76.81
69.28
57.76
52.54
46.26
32.29
29.92
26.92
23.52
18.95
5''
4''N
6''
3''
2''
1''
3p
2p
5p
6p
4'
3'
2'
1'
O
7
8
8a
O
2
O
6
5
4a
4
3
9
10
11
O
220 210 200 190 180 170 160 150 140 130 120 110 100 90 80 70 60 50 40 30 20 10 0
f1 (ppm)

KO-521C-1H

15

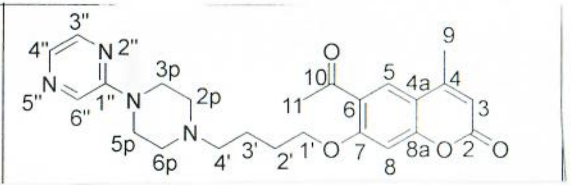
3"
4"
N 2"
3p
2p
10
5
4a
4
9
3
11
6
5"
6"
1"
N
5p
6p
4'
3'
2'
1'
O
7
8
8a
2

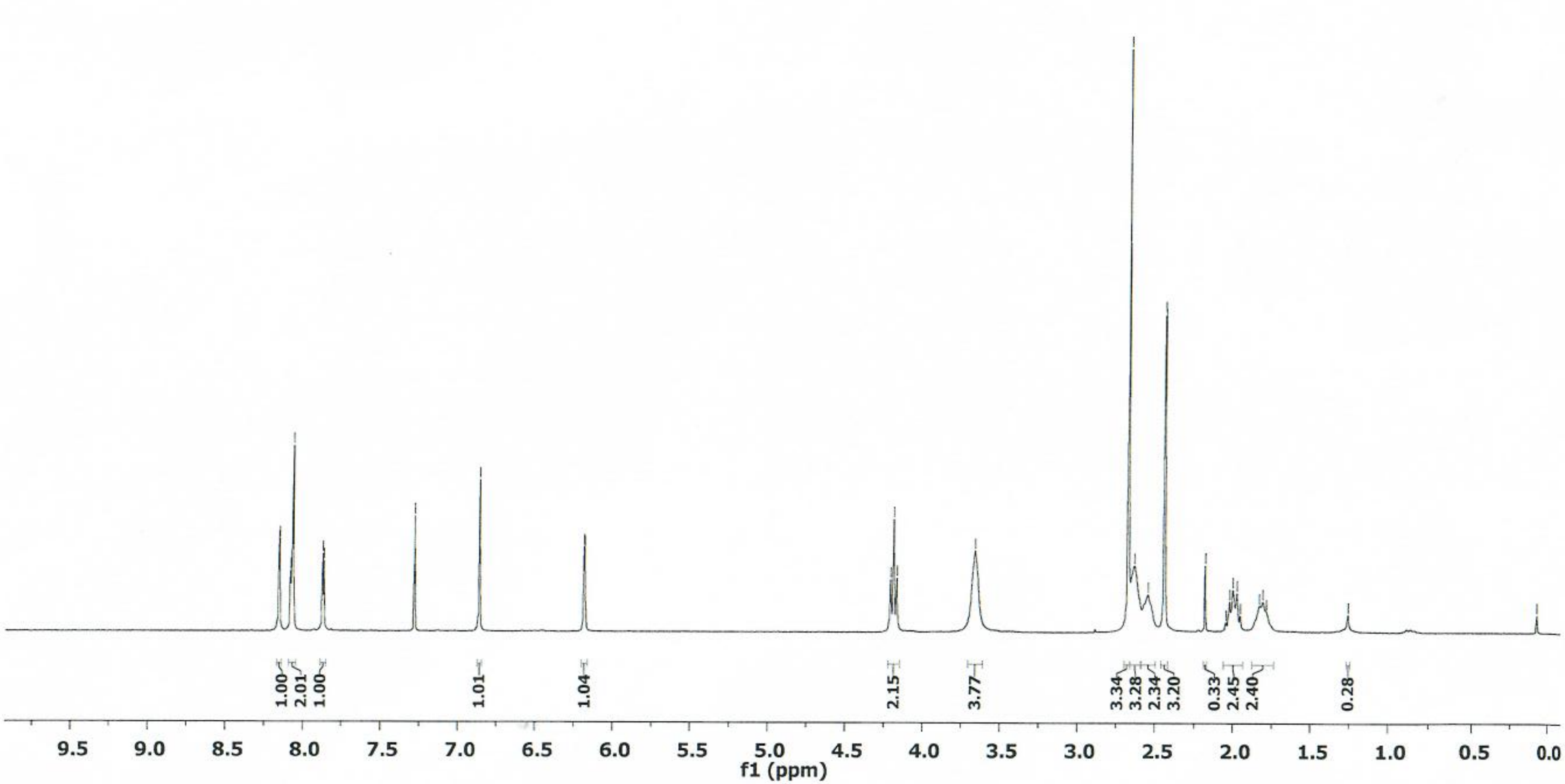
8.16
8.15
8.08
8.07
8.07
8.06
7.87
7.86
7.28
6.86
6.18
6.18
4.21
4.19
4.17
3.66
2.67
2.63
2.55
2.44
2.44
2.18
2.02
2.00
1.97
1.95
1.83
1.81
1.79
1.26
0.08
1.00
2.01
1.00
1.01
1.04
2.15
3.77
3.34
3.28
2.34
3.20
0.33
2.45
2.40
0.28
9.5 9.0 8.5 8.0 7.5 7.0 6.5 6.0 5.5 5.0 4.5 4.0 3.5 3.0 2.5 2.0 1.5 1.0 0.5 0.0
f1 (ppm)

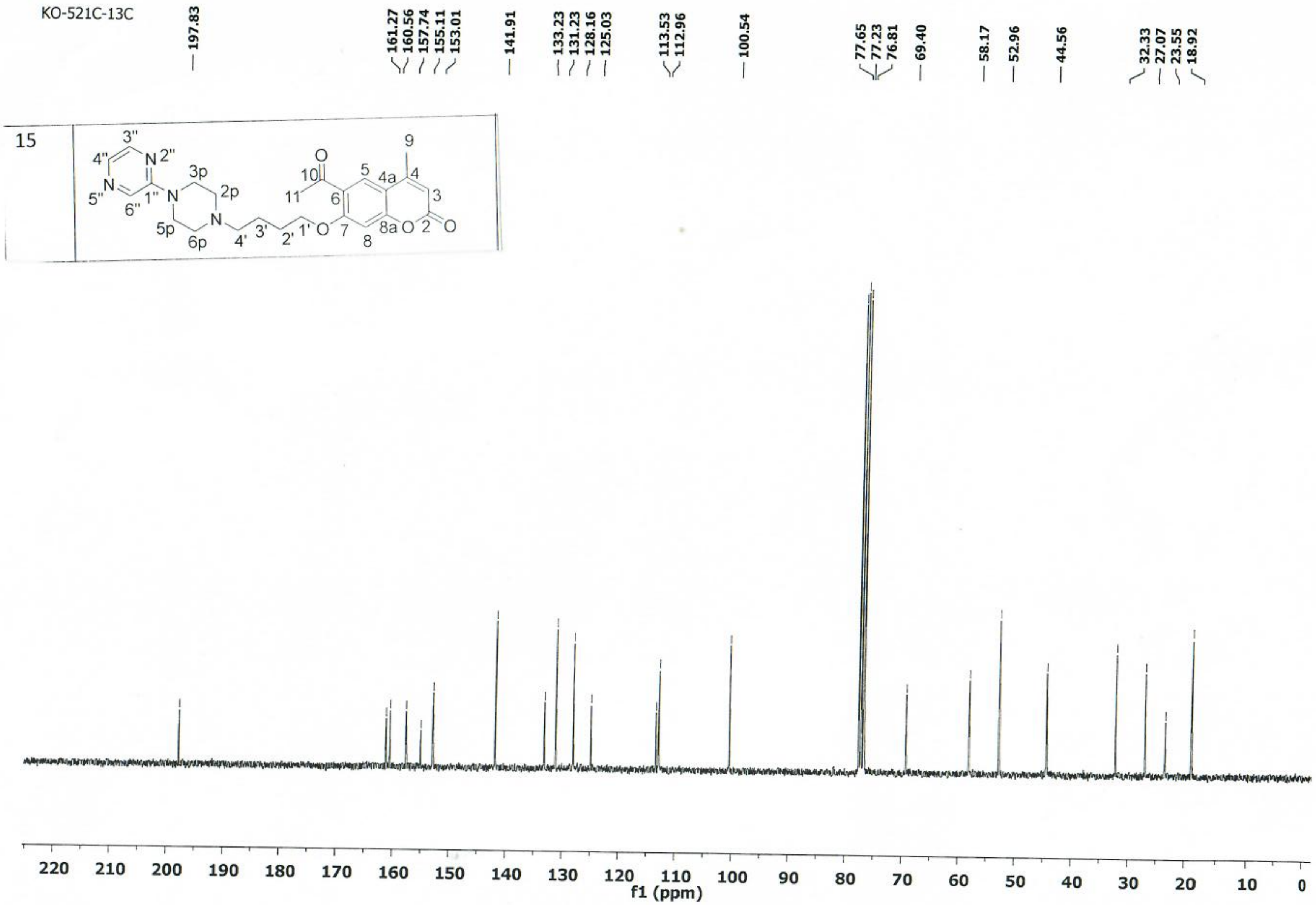

KO-521C-13C
197.83
161.27
160.56
157.74
155.11
153.01
141.91
133.23
131.23
128.16
125.03
113.53
112.96
100.54
77.65
77.23
76.81
69.40
58.17
52.96
44.56
32.33
27.07
23.55
18.92
15
220 210 200 190 180 170 160 150 140 130 120 110 100 90 80 70 60 50 40 30 20 10 0
f1 (ppm)
